# Noradrenergic terminal short-term potentiation enables modality-selective integration of sensory input and vigilance state

**DOI:** 10.1126/sciadv.abk1378

**Published:** 2021-12-17

**Authors:** Shawn R. Gray, Liang Ye, Jing Yong Ye, Martin Paukert

**Affiliations:** 1 Department of Cellular and Integrative Physiology, University of Texas Health Science Center at San Antonio, San Antonio, TX, USA.; 2 Joint UTSA/UTHSCSA Graduate Program in Biomedical Engineering, San Antonio, TX, USA.; 3 Department of Biomedical Engineering, University of Texas at San Antonio, San Antonio, TX, USA.; 4 Center for Biomedical Neuroscience, University of Texas Health Science Center at San Antonio, San Antonio, TX, USA.

## Abstract

Recent years have seen compelling demonstrations of the importance of behavioral state on sensory processing and attention. Arousal plays a dominant role in controlling brain-wide neural activity patterns, particularly through modulation by norepinephrine. Noradrenergic brainstem nuclei, including locus coeruleus, can be activated by stimuli of multiple sensory modalities and broadcast modulatory signals via axonal projections throughout the brain. This organization might suggest proportional brain-wide norepinephrine release during states of heightened vigilance. Here, however, we have found that low-intensity, nonarousing visual stimuli enhanced vigilance-dependent noradrenergic signaling locally in visual cortex, revealed using dual-site fiber photometry to monitor noradrenergic Ca^2+^ responses of astroglia simultaneously in cerebellum and visual cortex and two-photon microscopy to monitor noradrenergic axonal terminal Ca^2+^ dynamics. Nitric oxide, following *N*-methyl-d-aspartate receptor activation in neuronal nitric oxide synthase-positive interneurons, mediated transient acceleration of norepinephrine-dependent astroglia Ca^2+^ activation. These findings reveal a candidate cortical microcircuit for sensory modality-selective modulation of attention.

## Teaser

Sensory-driven cross-talk between interneurons and noradrenergic terminals conveys modality selectivity to neural modulation.

## INTRODUCTION

Patterns of neural activity within sensory cortices are determined by many behavioral, attentional, and environmental components in addition to the sensory information that is processed, with arousal playing an important role (*1*–*3*). Experimental evidence has accumulated that locomotion, a quantifiable form of active behavioral state, causes release of the neuromodulators acetylcholine and norepinephrine, resulting in considerable gain control over sensory processing in primary visual cortex (V1) (*4*–*10*). Noradrenergic signaling in the brain serves a broad range of functions from global energy mobilization to more regional effects, supporting working memory and attention (*3*, *11*–*13*). The locus coeruleus (LC), the main noradrenergic nucleus in the brainstem, with its projections throughout the forebrain and cerebellum (*14*) combined with its equally widespread, highly convergent afferent innervation pattern (*15*), supports global behavioral state and sensory input-dependent modulation (*16*). Projections from LC to cortical fields, such as prefrontal cortex and primary motor cortex, are segregated (*17*, *18*); nevertheless, individual LC neuron axons can cover brain-wide landscapes by branching to forebrain and cerebellum (*19*), suggesting that hardwired differences among LC projections may not be sufficient to account for selective modulation of noradrenergic signaling in individual regions of the brain. The possibility that norepinephrine release could be locally regulated has been considered (*20*), but, now, there is no direct experimental evidence whether and how this concept would be used during awake behavior.

Astroglia are nonexcitable cells with a ubiquitous presence throughout the central nervous system. They use dynamic changes of intracellular Ca^2+^ to respond to neurotransmitters and to modulate neuronal activity and behavior (*21*–*24*). Astrocyte Ca^2+^ signaling has been implicated in the modulation of diverse behaviors such as decision-making (*25*), memory acquisition (*26*–*28*), memory consolidation (*29*–*31*), and modulation of mechanosensation (*32*). In addition, chemogenetic enhancement of striatal astrocyte Ca^2+^ has led to hyperactivity-associated impairment of attention (*33*). Heightened states of vigilance elicited by arousal during locomotion cause widespread astroglia Ca^2+^ elevations in cerebellar Bergmann glia and cortical astrocytes (*34*–*36*), which are dependent on α_1_-adrenergic receptor activation (*36*, *37*) and intracellular Ca^2+^ release (*38*). The importance of noradrenergic signaling for arousal-associated astroglia Ca^2+^ elevations is further substantiated by their evolutionary conservation (*39*, *40*). Recent studies in mice have demonstrated that norepinephrine directly targets astroglia through α_1A_-adrenergic receptors on spinal cord astrocytes and cerebellar Bergmann glia (*32*, *41*), and Bergmann glia require these receptors for locomotion-induced Ca^2+^ elevations (*41*). Astroglia express a considerable number of receptors that are capable of elevating intracellular Ca^2+^; however, so far, noradrenergic signaling is the only signaling pathway that has been demonstrated to be required for awake behavior-triggered whole-cell astroglia Ca^2+^ elevations (*24*). Therefore, whole-cell astroglia Ca^2+^ dynamics in the context of heightened vigilance can serve as an accessible and reliable endogenous proxy readout to monitor noradrenergic signaling in awake behaving mice.

Here, we used dual-site fiber photometry to simultaneously monitor V1 astrocyte and cerebellar astroglia Ca^2+^ dynamics in awake behaving mice. We complemented these experiments with two-photon microscopy of Ca^2+^ dynamics in V1 and cerebellar LC terminals. Our findings revealed that low-intensity nonarousing visual stimulation enhanced vigilance-dependent norepinephrine release in V1 but not in the cerebellum. Local inhibition of *N*-methyl-d-aspartate (NMDA) receptors and nitric oxide (NO) synthesis in V1 reduced the potentiation and abolished the acceleration of noradrenergic signaling. Selective genetic deletion of NMDA receptor function in neuronal NO synthase (nNOS or NOS1)–positive neurons was sufficient to abolish the visual stimulation-induced acceleration of noradrenergic signaling. Our findings suggest that cortical circuit activity provides feedforward control of local noradrenergic tone, possibly contributing to mechanisms of bottom-up attentional deployment.

## RESULTS

### Nonarousing natural scene presentation potentiates vigilance-dependent astroglia Ca^2+^ activation selectively in V1

**Fig. 1.**
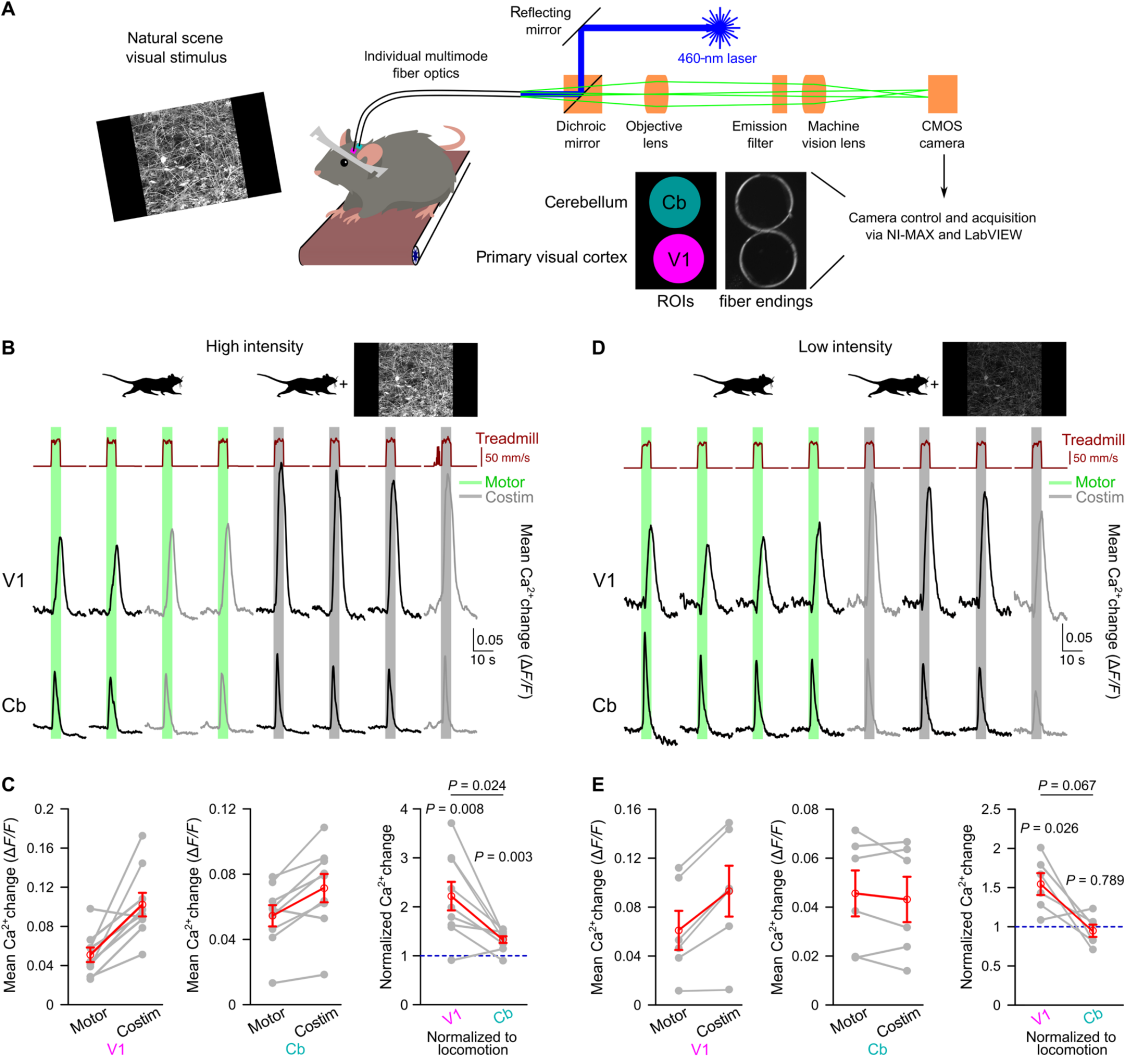
Nonarousing visual stimulation region specifically potentiates vigilance-dependent V1 astrocyte Ca^2+^ activation. (**A**) In vivo dual-site fiber photometry schematic for recording astroglia Ca^2+^ dynamics in cerebellum (Cb) and primary visual cortex (V1) from awake, head-fixed mice. (**B**) Ca^2+^ dynamics during enforced locomotion (green bars) or during simultaneous enforced locomotion and high-intensity (2.2 cd/m^2^) visual stimulation (gray bars) in V1 and Cb of *Aldh1l1-CreER^T2^*;Ai95 mice. Black traces represent mean Ca^2+^ responses within the regions of interest (ROIs) defined in (A). Gray traces represent voluntary locomotion contaminated trials; see Materials and Methods. (**C**) Left/middle: Population data of mean Ca^2+^ elevations from onset of locomotion to peak of locomotion or costimulation (Costim; high intensity) in V1 (left) and Cb (middle). Right: Ca^2+^ elevations during costimulation normalized to respective locomotion response in both regions (*n* = 9 mice). Gray lines, same mouse. Red symbols, means ± SEM. Repeated-measures analysis of variance (ANOVA) followed by Tukey-Kramer correction. Individual *P* values represent comparisons to 1 (blue dashed line), respectively. (**D**) Same as (B) but for low-intensity (0.3 cd/m^2^) visual stimulation. (**E**) Left/middle: Same as (C) left/middle but low intensity. Right: Same as (C) right but low intensity (*n* = 6 mice). Source data are provided as a source data file.

We applied fiber photometry simultaneously through chronic cranial windows above V1 and the cerebellar cortex to investigate whether and how global behavioral state-associated vigilance, heightened during locomotion, and visual input are integrated by noradrenergic signals (Fig. 1A and fig. S1). We chose crus I and lobulus simplex as anatomical regions of the cerebellum where the molecular mechanism of locomotion-induced Bergmann glia Ca^2+^ elevations has been found to depend on direct noradrenergic signaling (*41*), albeit the same behavioral context leads to very similar Bergmann glia Ca^2+^ elevations in other cerebellar regions, such as lobulus paramedianus (*36*) and vermis (*34*, *41*). Using dual-site fiber photometry on adult *Aldh1l1-CreER^T2^*;Ai95 mice that expressed the genetically encoded Ca^2+^ indicator GCaMP6f in most brain regions, including V1 and cerebellum, specifically in astroglia (fig. S2) (*42*), we compared Ca^2+^ dynamics in V1 astrocytes and cerebellar astroglia. The GCaMP6f fluorescence signals from both brain areas were monitored simultaneously by coupling an excitation laser beam into two individual fibers and projecting the fluorescence collected by the fibers onto the imaging chip of a complementary metal-oxide semiconductor (CMOS) camera (Fig. 1A). Two regions of interest (ROIs) for signals from V1 or cerebellum, respectively, could then be defined simultaneously and applied to all image frames. During voluntary or enforced locomotion, or other forms of arousal, astroglia respond to norepinephrine following activation of α_1_-adrenergic receptors (*36*–*38*). With cerebellar Bergmann glia, this locomotion-induced Ca^2+^ response is due to direct activation of α_1A_-adrenergic receptors (*41*). Combining visual stimulation with locomotion has been shown to potentiate locomotion-induced astrocyte Ca^2+^ activation in V1 (*36*), but it is not known whether potentiated V1 astrocyte activation is due to enhanced norepinephrine release or due to the integration of an independent signal by astrocytes. Furthermore, it is not known whether potentiation of astrocyte activation is confined to the visual system. Salient sensory stimuli of any modality can be arousing and activate LC (*16*). Consistent with a global enhancement of noradrenergic signaling, we found that natural scene visual stimulus exposure caused potentiated locomotion-induced Ca^2+^ elevations simultaneously in both V1 astrocytes and cerebellar astroglia (Fig. 1, B and C). Potentiation in V1 astrocytes was 3.7-fold stronger than in cerebellar astroglia (Fig. 1C), suggesting that a sensory-specific component may be involved. With attenuated salience of visual stimulation by reducing the brightness of the displayed natural scene, locomotion-induced cerebellar astroglia Ca^2+^ activation was no longer potentiated by visual stimulation. However, under presentation of the dimmed visual stimulus, a 33% potentiation of V1 astrocyte Ca^2+^ activation persisted (Fig. 1, D and E). In some experiments, comparing fluorescence responses to repeated locomotion events raised the concern that photobleaching could affect the measured signals over subsequent trials. However, the systematic comparison of the first responses that were not affected by voluntary locomotion to locomotion alone during experiments conducted for Fig. 1 with the respective last responses to locomotion alone did not reveal a consistent change in fluorescence (fig. S3). Our findings of a selective potentiation of norepinephrine-dependent V1 astrocyte Ca^2+^ elevations by low-intensity visual stimulation could be caused by either astrocytes integrating noradrenergic receptor activation with a local norepinephrine-independent circuit signal or the release of a local circuit factor that potentiates norepinephrine release. Therefore, the “astrocyte integration” model would predict that locomotion-induced LC terminal excitation in V1 and associated Ca^2+^ elevations would not be affected by low-intensity visual stimulation, whereas the “noradrenergic terminal integration” model would predict that low-intensity visual stimulation would potentiate locomotion-induced LC terminal Ca^2+^ elevations in V1.

### Blue-shifted visual stimulation is sufficient to potentiate vigilance-dependent astroglia Ca^2+^ activation and enables simultaneous two-photon microscopy

To differentiate between these models, noradrenergic axonal terminal Ca^2+^ dynamics with or without visual stimulation needed to be monitored. Because a considerable portion of terminal Ca^2+^ elevations are expected to originate from Ca^2+^ influx through voltage-gated Ca^2+^ channels, we chose the membrane-tethered Lck-GCaMP6f to observe Ca^2+^ dynamics in terminals with two-photon microscopy. The visual stimulation with a low-intensity natural scene, which was used for fiber photometry, would have had a considerable spectral overlap with the GCaMP6f detection in two-photon microscopy [compare “no filter” trace with green fluorescent protein (GFP) emission filter trace in fig. S4A]. To minimize cross-talk between natural scene visual stimulation and two-photon GCaMP6f detection, we covered the natural scene display with a KODAK Wratten 2 - no. 47 blue filter (fig. S4A). The low-intensity blue natural scene stimulus (blue stimulation) did not evoke any detectable signal with the GCaMP6f detector in mice without GCaMP6f expression (fig. S4B). Our finding of visual stimulation-induced V1-selective enhancement of vigilance-dependent astrocyte Ca^2+^ activation was replicable using blue stimulation in the dual-site fiber photometry setting (fig. S4C). Blue stimulation caused a 63% enhancement of V1 astrocyte locomotion-induced Ca^2+^ elevations with no effect on simultaneous cerebellar astroglia Ca^2+^ responses (fig. S4, C and D), demonstrating the viability of blue stimulation for extending our experimental paradigm to two-photon microscopy.

### Nonarousing natural scene presentation potentiates vigilance-dependent noradrenergic terminal Ca^2+^ elevations selectively in V1

**Fig. 2.**
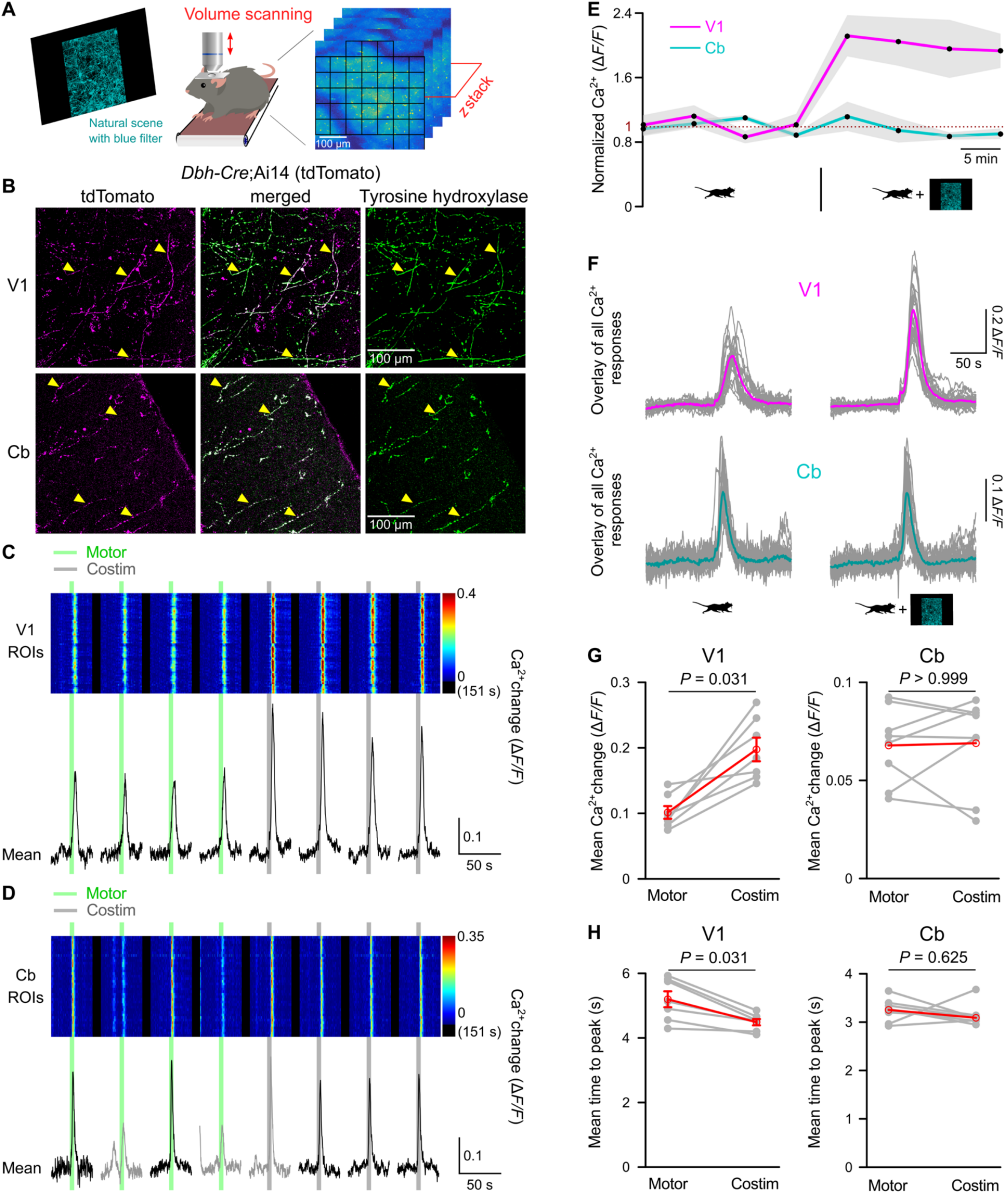
Visual cortex–specific potentiation of vigilance-dependent noradrenergic terminal Ca^2+^ elevations by nonarousing visual stimulation. (**A**) Schematic of two-photon volume Ca^2+^ imaging. (**B**) Top: V1 section from 8-week-old *Dbh*-*Cre*;Ai14 mouse with tdTomato fluorescence (magenta) and immunostained for tyrosine hydroxylase (green). Bottom: Same for Cb molecular layer from same mouse. (**C**) V1 noradrenergic terminal Ca^2+^ responses in *Dbh-Cre*;*Lck-GCaMP6f*^flox^ mice to enforced locomotion (green bars) or costimulation [low intensity, blue filtered (0.1 cd/m^2^); gray bars]. Pseudo-colored plot represents Ca^2+^ changes in ROIs schematized in (A). Black traces represent mean Ca^2+^ change in all ROIs. (**D**) Same for Cb molecular layer noradrenergic terminals. Gray traces represent voluntary locomotion contaminated trials. (**E**) Normalized means ± SEM (gray shading) of population Ca^2+^ dynamics in (C) and (D) (normalized to respective average locomotion trials). (**F**) Overlay of stimulus-aligned Ca^2+^ fluorescence traces in response to locomotion (left), costimulation (right), in V1 (top) or Cb (bottom). Magenta or cyan traces represent mean in V1 or Cb, respectively. (**G**) Chained-dot plots compare mean Ca^2+^ change during 10 s from onset of locomotion between locomotion and costimulation trials (V1, *n* = 7 mice; Cb, *n* = 8 mice). Gray lines, same mouse. Red symbols, means ± SEM or median. (**H**) Mean time to peak of the same data analyzed in (G). Wilcoxon signed-rank tests followed by Bonferroni correction. Source data are provided as a source data file.

For imaging locomotion-induced noradrenergic terminal Ca^2+^ dynamics through chronic cranial windows, we used volume scanning of Lck-GCaMP6f, which was exclusively expressed in noradrenergic neurons and their axons by restricting recombination of *Lck-GCaMP6f* ^flox^ (*42*) to Cre recombinase activity in dopamine-β-hydroxylase (*Dbh*)–*Cre* mice (Fig. 2, A and B) (*43*). The overlap of Cre-dependent expression of tdTomato in cerebellar molecular layer and V1 of *Dbh-Cre*;Ai14 mice with the catecholaminergic neuron marker tyrosine hydroxylase confirmed specific recombination in noradrenergic neurons (Fig. 2B). Because the membrane-tethered Lck-GCaMP6f signal did not allow the reliable identification of individual axons and terminal varicosities, we applied an unbiased checkerboard pattern of ROIs, each containing responsive clusters of axons and terminals (Fig. 2A), visualized individual ROIs’ Ca^2+^ dynamics (Fig. 2, C and D), and analyzed the mean of all ROI responses. This approach prevents that the analysis could be skewed by intense regional responses and the visualization would allow for the detection of regional modulation of Ca^2+^ responses. Locomotion reliably induced transient Ca^2+^ elevations in V1 as well as in cerebellar noradrenergic terminals (Fig. 2, C and D) with consistently faster kinetics in cerebellum [time from onset of locomotion to peak: V1, 5.2 ± 0.2 s versus Cb, 3.2 ± 0.1 s; *t*(13) = 7.941, *P* < 0.001; *n* = 7 and 8, respectively; unpaired, two-tailed Student’s *t* test]. In V1, the responses were uniformly potentiated approximately twofold and were accelerated by concomitant blue stimulation (Fig. 2, C and E to H). In contrast, blue stimulation did not affect locomotion-induced Ca^2+^ elevations in cerebellar noradrenergic terminals (Fig. 2, D to H). These findings were consistent with the noradrenergic terminal integration model, suggesting that potentiation of norepinephrine release was restricted to areas of ongoing visual processing.

### Visual input-induced potentiation of vigilance-dependent V1 astrocyte Ca^2+^ activation is short-lived and confined to individual trials

**Fig. 3.**
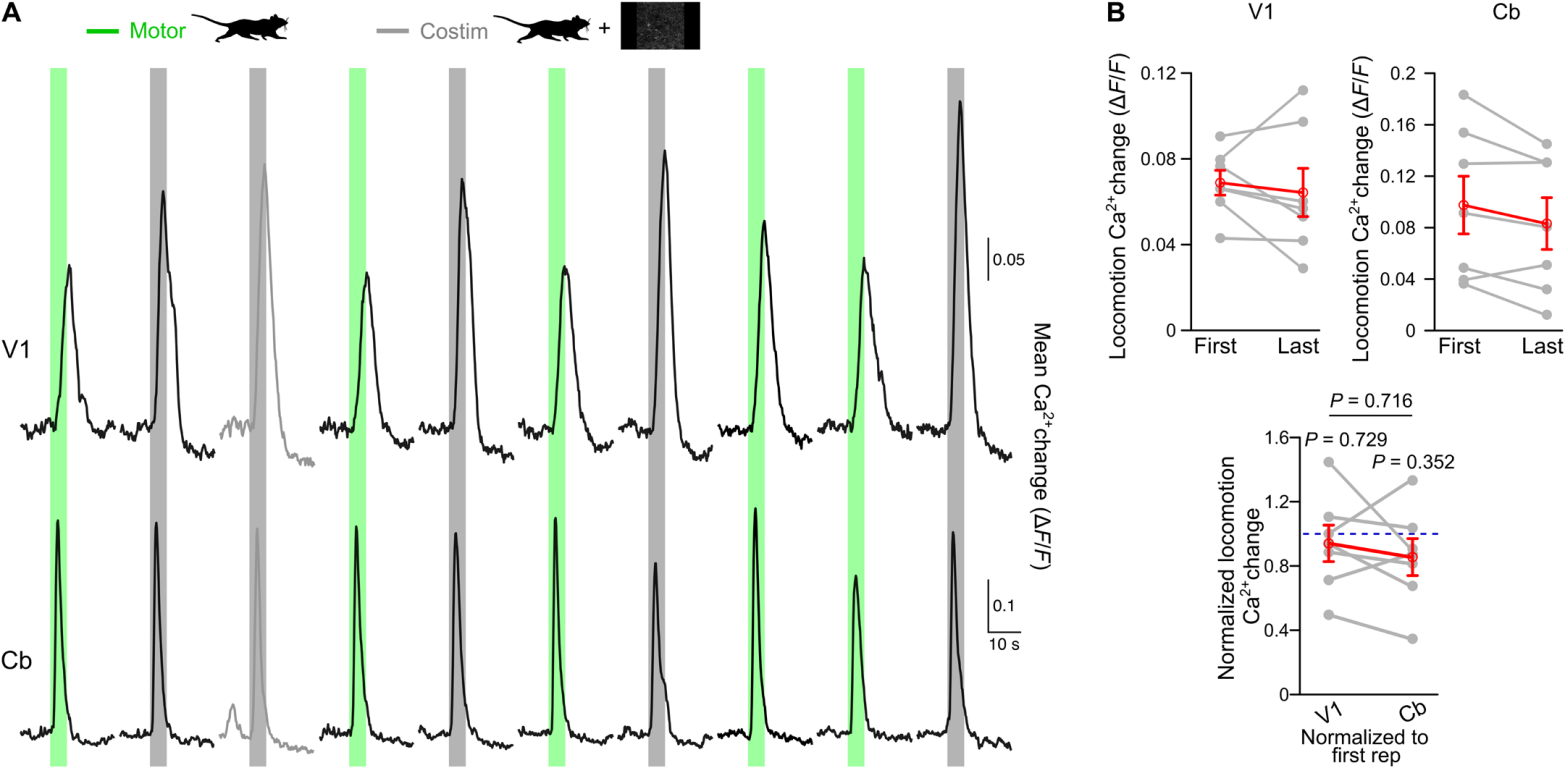
Sensory stimulus-evoked potentiation of noradrenergic terminals is short-lived. (**A**) Astroglia Ca^2+^ dynamics recorded with dual-site fiber photometry in *Aldh1l1-CreER^T2^*;Ai95 mice during a pseudo-randomized (five each) order of locomotion-alone (green bars) or simultaneous locomotion and low-intensity (0.3 cd/m^2^) visual stimulation trials (gray bars) in both regions. Black traces represent mean Ca^2+^ responses in V1 (top) or Cb (bottom). Traces in shaded gray represent voluntary locomotion contaminated trials. (**B**) Top: If the first “uncontaminated” locomotion-alone trial happened before the first costimulation trial, then its mean Ca^2+^ change in V1 (left) or Cb (right) was compared to the last locomotion-alone trial. Bottom: Ca^2+^ elevations of the last locomotion-alone trial normalized to respective first uncontaminated locomotion-alone trial in both regions (*n* = 7 mice). Gray lines connect values from the same mouse. Red symbols indicate means ± SEM. Statistical analysis used repeated-measures ANOVA followed by Tukey-Kramer correction. Individual *P* values for normalized Ca^2+^ changes in V1 or Cb represent comparisons to 1 (blue dashed line), respectively. Source data are provided as a source data file.

In a first approach toward understanding the underlying mechanism of visual stimulation-induced potentiation of noradrenergic terminals, we asked the question how long this potentiation lasts. To distinguish short-term potentiation, which would last for the period of visual exposure without affecting consecutive locomotion-alone events, from long-term potentiation, where locomotion-alone events were dependent on prior visual exposure history, we applied a pseudo-randomized order of five locomotion-alone trials and five trials with coinciding locomotion and low-intensity natural scene presentation (Fig. 3A). Comparing the first locomotion-alone–induced Ca^2+^ elevation before any exposure to visual stimulation with the last locomotion-alone–induced response following at least one visual stimulation revealed no difference in either V1 astrocytes or in cerebellar astroglia (Fig. 3B). Both regions exhibited a trend toward a gradual reduction of locomotion-induced astroglia Ca^2+^ elevations indicating that potentiation of V1 astrocyte responses by low-intensity visual stimulation was confined to individual trials.

### Visual input-induced potentiation of vigilance-dependent V1 noradrenergic terminal Ca^2+^ elevations requires NMDA receptor activation and NO production

**Fig. 4.**
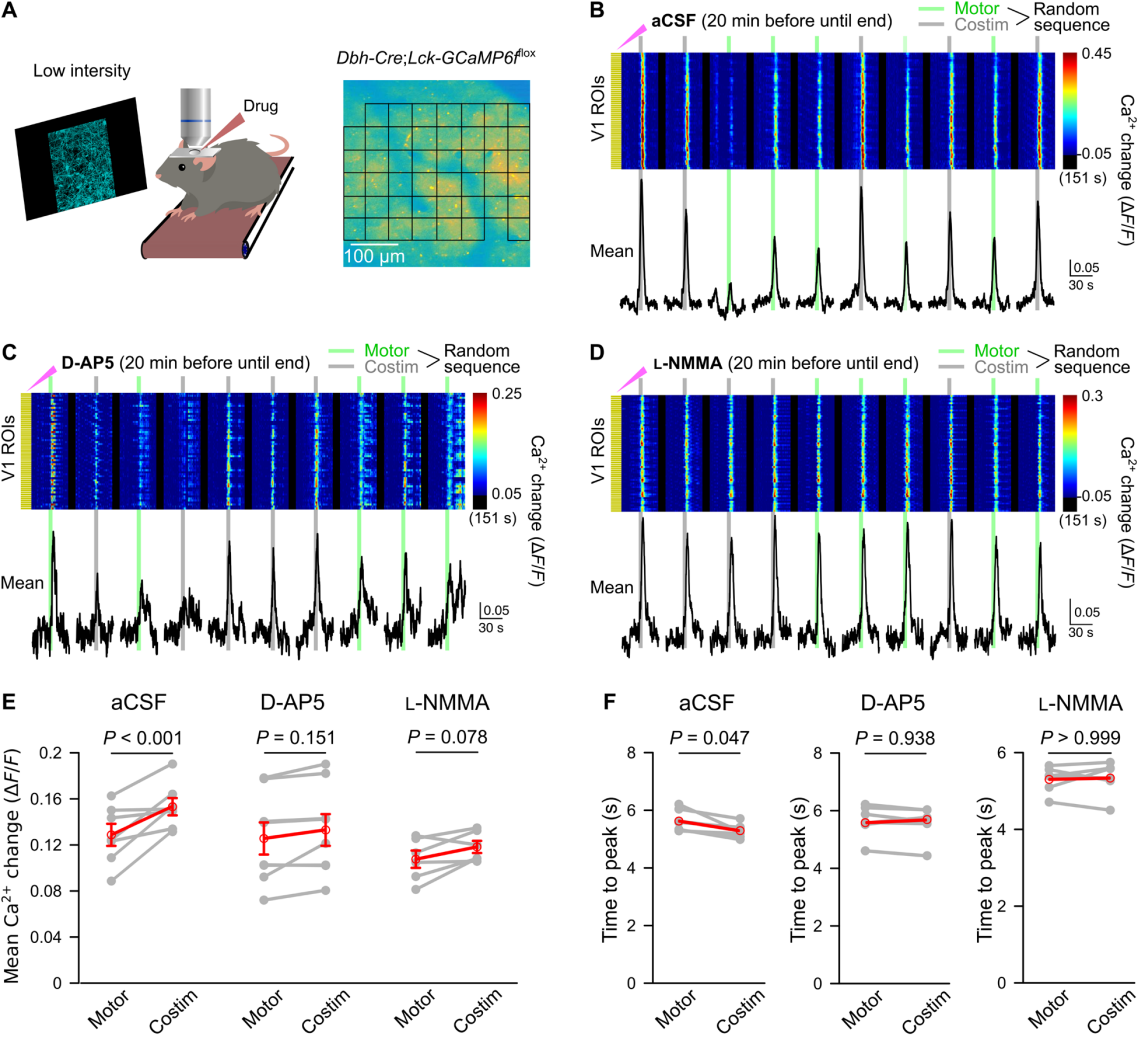
NMDA receptors and NO mediate local, sensory stimulus-induced enhancement and acceleration of noradrenergic terminal Ca^2+^ elevations in V1. (**A**) Experimental design as for Fig. 2 and pseudo-randomized stimulation as for Fig. 3. Incubation with drugs in artificial cerebrospinal fluid (aCSF) started 20 min before imaging (solution replacement every 10 min). (**B**) Ca^2+^ responses to enforced locomotion (green bars) or simultaneous enforced locomotion and blue stimulation (gray bars) in aCSF. Pseudo-colored plot represents Ca^2+^ changes in individual ROIs, and black traces represent mean Ca^2+^ change in all ROIs. (**C**) Same as (B) with NMDA receptor antagonist D-AP5 (1 mM). (**D**) Same as (B) with NOS inhibitor l-NMMA acetate (600 μM). (**E**) Chained-dot plot to compare mean Ca^2+^ change during 10 s from onset of locomotion in all noradrenergic terminal ROIs between locomotion and costimulation trials from all mice locally exposed to aCSF (left, *n* = 7 mice), D-AP5 (middle, *n* = 8 mice), and l-NMMA acetate (right, *n* = 6 mice). Gray lines, same mouse. Red symbols, means ± SEM. Repeated-measures ANOVA for three groups, followed by Tukey-Kramer correction. (**F**) Same as (E) but comparing mean time to peak. Red symbols, median. Wilcoxon signed-rank tests followed by Bonferroni correction. Source data are provided as a source data file.

Retrograde labeling revealed anatomical segregation of LC neuron projections to various cortical areas (*17*), raising the possibility that short-lived potentiation of noradrenergic neuron activation could occur in LC. Alternatively, a potential mechanism for short-term axonal terminal potentiation of norepinephrine release could happen through activity-dependent activation of NMDA receptors in the local circuit and subsequent production of NO, a pathway that has been found to enhance norepinephrine release in the synaptosome preparation (*44*) and in laterodorsal tegmentum upon administration of NMDA (*45*). To test the hypothesis of visual stimulation-induced local NMDA receptor activation and NO production, we investigated the effect of local drug application on V1 noradrenergic terminal Ca^2+^ elevations evoked by pseudo-randomly ordered trials of locomotion alone or locomotion combined with blue stimulation (Fig. 4). Despite the invasiveness of local pharmacology experiments when compared to imaging through chronic cranial windows, blue stimulation induced a 19% increase in locomotion-induced V1 noradrenergic terminal Ca^2+^ elevations (Fig. 4, B and E) and accelerated the Ca^2+^ rise under control conditions (Fig. 4F). Following local preincubation of the V1 surface with the NMDA receptor antagonist D-2-amino-5-phosphonovalerate (D-AP5; Fig. 4C) or with the NO synthase inhibitor NG-monomethyl-l-arginine acetate (l-NMMA; Fig. 4D), there remained only a trend toward a potentiating effect of visual stimulation on the amplitude of the locomotion-induced Ca^2+^ elevation (Fig. 4E), and its accelerating effect on the Ca^2+^ rise was abolished (Fig. 4F). Neither D-AP5 nor l-NMMA affected noradrenergic terminal Ca^2+^ elevations by locomotion alone (fig. S5A). Together, these findings suggest that NMDA receptor-dependent NO synthesis following nonarousing visual stimulation contributes to temporarily potentiating vigilance-dependent norepinephrine release.

### Visual input-induced potentiation of vigilance-dependent V1 astrocyte Ca^2+^ activation mirrors the pharmacological sensitivity of noradrenergic terminals

**Fig. 5.**
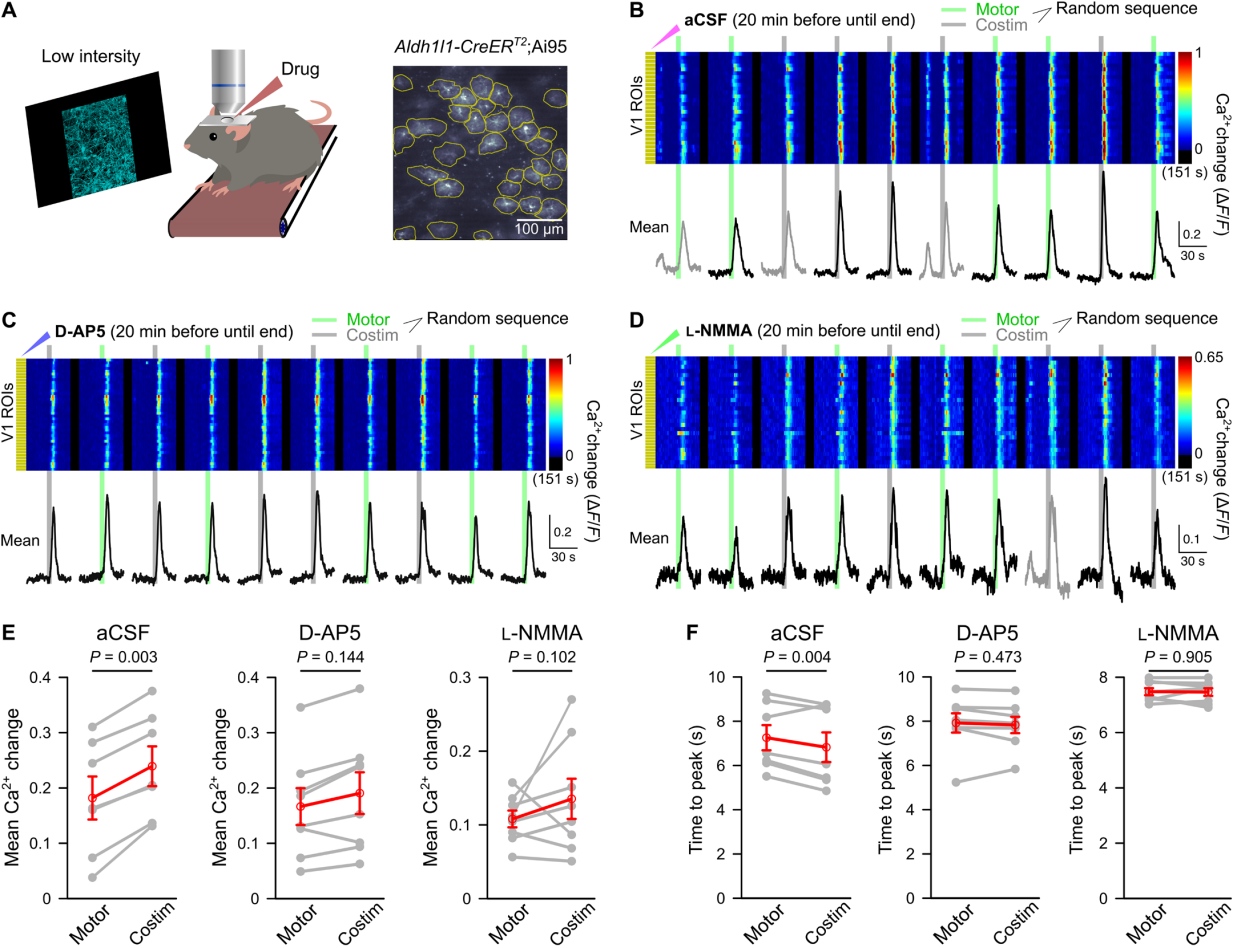
Sensory stimulus-evoked potentiation of astrocyte Ca^2+^ elevations requires NMDA receptor activation and NO. (**A**) Schematic illustrating same experimental design as described for Fig. 4 using *Aldh1l1-CreER^T2^*;Ai95 mice. (**B**) Ca^2+^ responses to enforced locomotion (green bars) or simultaneous enforced locomotion and blue stimulation (gray bars). The surface of V1 was incubated in aCSF from 20 min before until the end of the experiment (aCSF was replaced every 10 min). Pseudo-colored plot represents Ca^2+^ changes in individual ROIs, and black traces represent mean Ca^2+^ change in all ROIs. (**C**) Same design as (B) but incubation with NMDA receptor antagonist D-AP5 (1 mM). (**D**) Same design as (B) but incubation with NOS inhibitor l-NMMA acetate (600 μM). (**E**) Chained-dot plot to compare mean Ca^2+^ change during 10 s from onset of locomotion in all astrocytes between locomotion-alone and costimulation trials from all mice locally exposed to aCSF (left, *n* = 7 mice), D-AP5 (middle, *n* = 8 mice), and l-NMMA acetate (right, *n* = 8 mice). Gray lines connect values from the same mouse. Red symbols indicate means ± SEM. Statistical analysis used repeated-measures ANOVA for three groups, followed by Tukey-Kramer correction. (**F**) Same as (E) but comparing mean time to peak. Gray lines connect values from the same mouse. Red symbols indicate means ± SEM. Statistical analysis used repeated-measures ANOVA for three groups, followed by Tukey-Kramer correction. Source data are provided as a source data file.

It is possible that vigilance-dependent V1 astrocyte Ca^2+^ elevations were enhanced by visual stimulation through an additional local mechanism directly acting on astrocytes independent of enhanced norepinephrine release. Therefore, we investigated whether V1 local pharmacological inhibition of NMDA receptors and NO production had similar effects on visual input-induced potentiation of vigilance-dependent astrocyte Ca^2+^ activation as it had on noradrenergic terminals. For studying the effect of local drug applications on Ca^2+^ dynamics in V1 astrocytes, we used volume scanning two-photon microscopy and defined individual ROIs on the basis of visually identified astrocytes (Fig. 5A). The V1 astrocyte Ca^2+^ activation during pseudo-randomly ordered sequences of locomotion-alone trials or locomotion trials combined with blue stimulation was potentiated by 32% (Fig. 5, B and E) and was accelerated by visual stimulation (Fig. 5F). Like with our findings with noradrenergic terminals, following local preincubation of the V1 surface with D-AP5 (Fig. 5C) or with l-NMMA (Fig. 5D), there remained only a trend toward potentiation of the response amplitude by visual input (Fig. 5E), and its accelerating effect on the Ca^2+^ rise was completely abolished (Fig. 5F). Some mice still showed robust potentiation of locomotion-induced V1 astrocyte Ca^2+^ activation despite inhibition of NO production; however, potentiation of responses to low-intensity visual stimulation was also observed in cerebellar astroglia of some mice (Figs. 1E and fig. S4D), suggesting that those mice were more sensitive to global arousal. Neither D-AP5 nor l-NMMA affected astrocyte Ca^2+^ activation by locomotion alone (fig. S5B). The similarity of the pharmacological profiles of potentiation of V1 astrocyte and noradrenergic terminal vigilance-dependent Ca^2+^ elevations by visual input implies that NO potentiates vigilance-dependent norepinephrine release and astrocytes reliably track and report changes in norepinephrine release.

### Visual input-induced activation of NMDA receptors in nNOS^+^ interneurons contributes to potentiation of noradrenergic signaling in V1

**Fig. 6.**
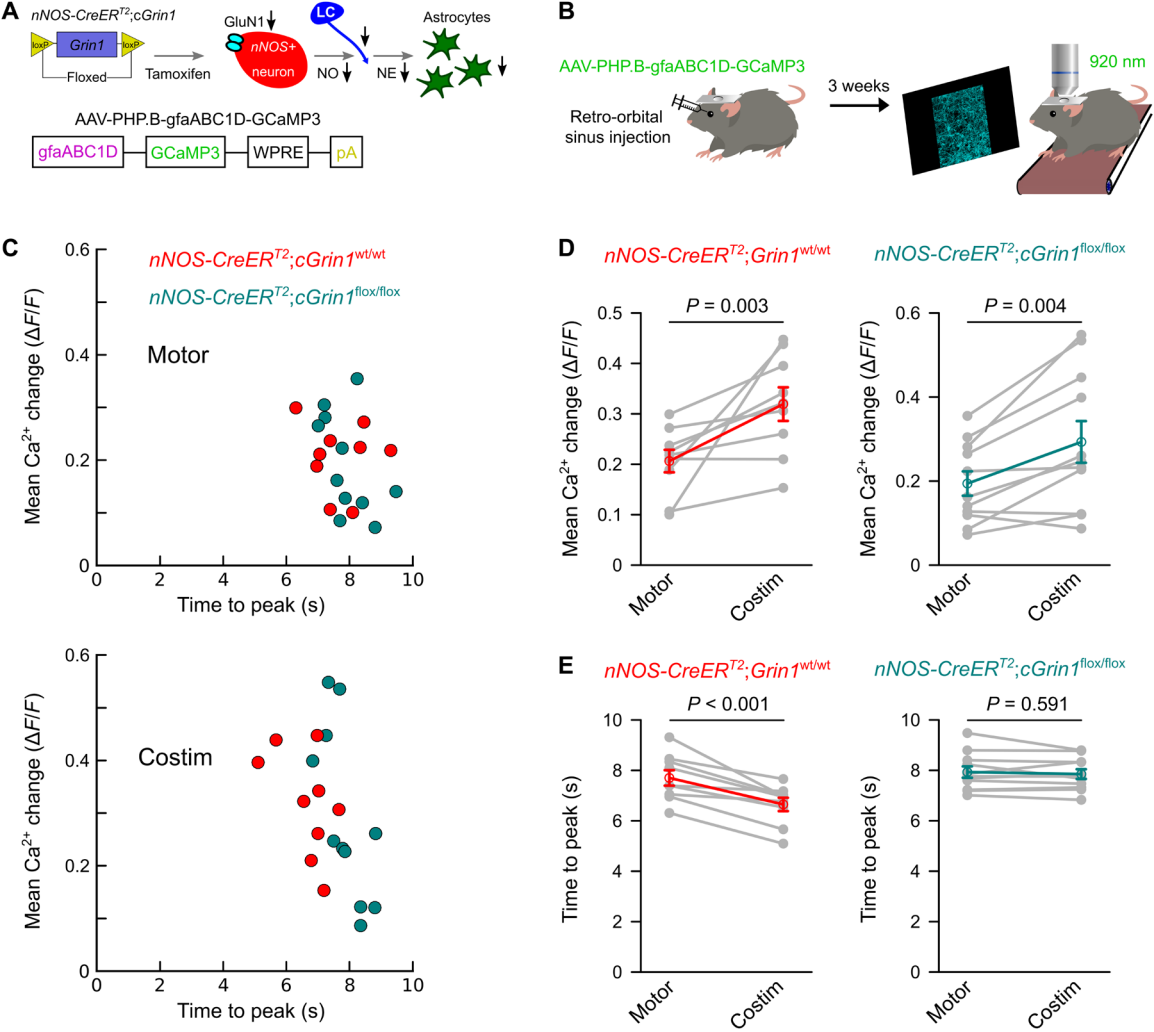
Induced knockout of GluN1 receptors on nNOS^+^ interneurons impairs the acceleration of noradrenergic signaling by visual input. (**A**) Schematic diagram of generating *nNOS-CreER^T2^*;c*Grin1*^flox/flox^ mice and AAV-PHP.B-gfaABC1D-GCaMP3. (**B**) Schematic of viral vector delivery by retro-orbital injection for in vivo astroglia Ca^2+^ imaging. (**C**) Population data with two-dimensional plots visualizing the mean Ca^2+^ change (*y* axis) and mean time to peak (*x* axis) for *nNOS-CreER^T2^*;c*Grin1*^wt/wt^ (red) and *nNOS-CreER^T2^*;c*Grin1*^flox/flox^ (green) mice during locomotion-alone trials (Motor) and costimulation trials (Costim). (**D**) Chained-dot plot to compare mean Ca^2+^ change between locomotion-alone and costimulation trials in *nNOS-CreER^T2^*;c*Grin1*^wt/wt^ and *nNOS-CreER^T2^*;c*Grin1*^flox/flox^ mice [*n* = 9 fields of view (FOVs) from three mice and *n* = 11 FOVs from four mice, respectively]. Gray lines connect values from the same mouse. Red and green symbols indicate means ± SEM. Statistical analysis used repeated-measures ANOVA for two groups, followed by Tukey-Kramer correction. (**E**) Same as (D) but comparing mean time to peak. Gray lines connect values from the same mouse. Red and green symbols indicate means ± SEM. Statistical analysis used repeated-measures ANOVA for two groups, followed by Tukey-Kramer correction. Source data are provided as a source data file.

nNOS^+^ interneurons represent a sparse population of interneurons with a dense network of long-ranging axonal projections within the cerebral cortex, and they express nNOS within their axonal projections (*46*, *47*). Thus, they represent a cellular candidate for visual input-induced, NMDA receptor-dependent NO production within V1. Because, with pharmacological approaches, it is not possible to distinguish between NMDA receptors on interneurons, principal neurons, glial cells, and endothelial cells, we used a genetic approach to test the hypothesis that NMDA receptor function in nNOS^+^ interneurons was required for visual input-induced NO production and subsequent short-term potentiation of norepinephrine release and astrocyte Ca^2+^ activation (Fig. 6A). We generated transgenic mice with loxP-flanked alleles of the gene *Grin1*, which encodes the critical subunit 1 of the NMDA type ionotropic glutamate receptor (GluN1) (*48*), and with one allele carrying *CreER^T2^* knocked into the nNOS gene location (Fig. 6A) (*49*). Three weeks after tamoxifen induction of Cre-dependent *Grin1* deletion in nNOS^+^ interneurons, we delivered adeno-associated virus (AAV) particles, which used the blood-brain-barrier penetration facilitating capsid PHP.B (*50*) and had the expression of GCaMP3 controlled by the astroglia-specific promoter gfaABC1D (Fig. 6A) (*51*), to the retro-orbital venous sinus. Three weeks later, the V1 astrocyte Ca^2+^ activation during pseudo-randomly ordered sequences of locomotion-alone trials or locomotion trials combined with blue stimulation were assessed in *Grin1*^flox/flox^ mice and wild-type littermates using two-photon volume scanning (Fig. 6B). Selective deletion of NMDA receptor function in nNOS^+^ interneurons did not affect the amplitude of visual stimulation-induced enhancement of vigilance-dependent V1 astrocyte Ca^2+^ activation (Fig. 6D), but it was sufficient to completely abolish the visual stimulation-induced acceleration of the astrocyte Ca^2+^ rise (Fig. 6E). Conditional deletion of NMDA receptor function had no effect on size or kinetics of astrocyte Ca^2+^ activation by locomotion alone (fig. S6). These findings suggest that NMDA receptor-dependent nNOS activation and NO synthesis contribute to short-lived potentiation of vigilance-dependent norepinephrine release and consecutively accelerated astrocyte Ca^2+^ activation.

## DISCUSSION

Our findings demonstrate that visual cortex noradrenergic terminals integrate information about visual stimulation and the global vigilance state. As long as the visual stimulation is nonarousing, the potentiation of vigilance-dependent noradrenergic terminal Ca^2+^ elevation amplitude and kinetics are modality-specific and restricted to V1. The integration is accomplished, in part, by local circuit activity-dependent activation of NMDA receptors on nNOS^+^ interneurons and production of NO, leading to increased vigilance-dependent terminal Ca^2+^ elevations and norepinephrine release. Astrocytes reliably track size and kinetics of noradrenergic signals. This implies that other direct targets of norepinephrine within the local circuitry, depending on the adrenergic receptors they express, obtain integrated information about vigilance state and sensory input, and astroglia may rather serve as amplifiers. It further emphasizes the potentially important role of astroglia in diseases with alterations in noradrenergic signaling, such as neurodegenerative diseases (*52*, *53*) or diseases of hyperactivity and deficits in attention (*54*).

V1 astrocyte Ca^2+^ responses to visual stimulation in awake mice have been studied before, under various experimental conditions. It was found that light-emitting diode (LED) visual stimulation alone was not sufficient to evoke whole-astrocyte Ca^2+^ elevations in V1 of awake mice while at rest (*36*). Consistent with this finding, using moving gratings as visual stimuli and despite analyzing subcellular Ca^2+^ dynamics, one study barely found any V1 astrocyte Ca^2+^ elevation beyond spontaneous microdomain Ca^2+^ events (*55*). In another study, which used similar visual stimuli, weak somatic Ca^2+^ elevations, which were not orientation-selective, were observed; however, visual stimulation-associated responses, which might have been expected to occur in the astrocyte processes, were not detected (*56*). In contrast, concomitant LED visual stimulation with locomotion has been demonstrated to potentiate V1 astrocyte Ca^2+^ activation compared to locomotion-alone trials (*36*). However, this study could not distinguish between visual stimulation, causing local V1-selective potentiation of vigilance-dependent astrocyte Ca^2+^ activation, or whether it led to enhanced activation of LC and consecutively enhanced global arousal. It was found that, when mice were exposed to moving gratings while running, their V1 astrocyte Ca^2+^ responses correlated with pupil size, indicating that they depended on arousal (*55*). Because the responses were also orientation-selective, the authors proposed a model where astrocytes would sense a neurotransmitter other than norepinephrine, locally released by visual stimulation, and integrate this signal with the vigilance-dependent norepinephrine (*55*). We considered this possibility earlier here as the astrocyte integration model. In this study, simultaneous dual-fiber photometry of V1 astrocyte and cerebellar astroglia Ca^2+^ dynamics allowed us to directly demonstrate that nonarousing natural scene visual stimulation potentiated vigilance-dependent Ca^2+^ activation selectively in V1 but not in the cerebellum. To directly test the astrocyte integration hypothesis, we imaged vigilance-dependent Ca^2+^ elevations in noradrenergic terminals in V1 and cerebellum. Contrary to this model, we found that natural scene stimulation induced a reliable potentiation of the amplitude and accelerated vigilance-dependent Ca^2+^ elevations in noradrenergic terminals. In direct disaccord with the astrocyte integration model, the potentiation of noradrenergic terminal responses was selective for V1 and absent in the cerebellum. On the basis of these findings and the close similarity between the pharmacological sensitivities of visual input-dependent potentiation of V1 noradrenergic terminal and astrocyte Ca^2+^ elevations discussed later, we propose the noradrenergic terminal integration model as a cortical circuit property to integrate sensory input and global vigilance state in a modality-selective manner.

The noradrenergic terminal integration previously unknown form of plasticity represents a form of short-term potentiation, because the facilitation of norepinephrine release lasted for less than the approximately 3.3-min interval between stimulation trials in our paradigm. Therefore, the potentiation was restricted to individual trials and enabled reliable encoding of information about visual input and heightened vigilance state by V1 noradrenergic signaling. Short-term presynaptic regulation of neurotransmitter release is usually attributed to activity patterns supportive of Ca^2+^ buildup in the presynaptic axon terminal or to retrograde messengers (*57*). In addition, axonal excitability can be modulated by axonal neurotransmitter-gated ion channels with modulation of release from glutamatergic axons in diverse brain regions representing the most prevalent example (*58*–*64*). Norepinephrine release can be facilitated by NO in a cortical synaptosome preparation or in the laterodorsal tegmentum of anesthetized rats upon NMDA receptor agonist application (*44*, *45*). We found that acute pharmacological inhibition of NMDA receptors or NO synthesis in V1 weakened natural scene visual stimulation-induced potentiation of vigilance-dependent Ca^2+^ response amplitude and abolished acceleration of those responses both in noradrenergic terminals and in astrocytes. In addition to whole-cell Ca^2+^ responses, astroglia undergo spontaneous subcellular Ca^2+^ dynamics restricted to the microdomain level, sometimes extending to process branches (*38*), and many different neurotransmitter receptors and ion channels contribute to these diverse signaling events (*21*, *65*). Because Ca^2+^ sensing is a nonlinear process, it is conceivable that enhancement of one or more types of microdomain events during visual input could contribute to the potentiation of vigilance-dependent V1 astrocyte whole-cell Ca^2+^ elevations. While our data cannot exclude the contribution of an additional signal acting directly on astrocytes, the pharmacological sensitivity of the effects of visual input on astrocytes were precisely mirrored by the pharmacological sensitivity of the effects of visual input on noradrenergic terminals. Together with the prior observation that visual input has barely any effect on V1 astrocyte Ca^2+^ dynamics in awake mice at rest, these findings suggest that the vast majority of integration of information about visual input and vigilance state happens at noradrenergic terminals. The inhibition of visual input-induced potentiation of the amplitude of vigilance-dependent Ca^2+^ elevations was incomplete. This could either suggest that there is another neurotransmitter acting on noradrenergic terminals or it could reflect the challenges that intact blood-perfused brain tissue poses to in vivo pharmacology experiments.

To identify the cellular and molecular source of NO and thereby better define this previously unknown circuit motif, we set out to test the hypothesis that NO was produced by nNOS, the most abundant NO synthase in the central nervous system (*66*), following activation of NMDA receptors on interneurons, which are defined by the expression of nNOS (*46*, *47*). Because nNOS activation is dependent on NMDA receptor-mediated elevation of Ca^2+^/calmodulin (*66*, *67*), we conditionally deleted GluN1 (*48*), the essential subunit of NMDA receptors, from nNOS^+^ interneurons (*49*). The effect on visual input-induced potentiation of vigilance-dependent V1 astrocyte Ca^2+^ activation was similar but less complete than the pharmacological inhibition of NMDA receptors or NO synthase. The kinetic portion of potentiation of Ca^2+^ responses of noradrenergic terminals and astrocytes was consistently more sensitive to pharmacological and to genetic manipulations than the amplitude portion. Therefore, it is likely that a considerable potentiation is required before the kinetics are affected. In this regard, the observation that the acceleration of the astrocyte Ca^2+^ rise was abolished completely but the amplitude was not affected by selective genetic deletion of NMDA receptor function in nNOS^+^ interneurons could be explained by incomplete recombination in our tamoxifen-inducible gene deletion strategy. Alternatively, some of the NO could also originate from endothelial NOS, which is dependent on endothelial NMDA receptor activation (*68*, *69*) and has been implicated in sensory-driven Ca^2+^ elevation in astrocyte endfeet, which cover the vasculature (*70*). In addition, NMDA receptors could also be activated as presynaptic receptors (*71*, *72*) directly on noradrenergic terminals (*73*), as it was proposed but not yet directly demonstrated for cholinergic terminals (*74*, *75*). However, pharmacological inhibition of NMDA receptors did not appear to have a more complete inhibitory effect on visual input-induced potentiation of vigilance-dependent V1 noradrenergic terminal and astrocyte Ca^2+^ responses than inhibition of NO production. Recent work has demonstrated that locomotion triggers a Ca^2+^ elevation in cortical nNOS^+^ interneurons, which was found to be sufficient to cause arterial vasodilation (*76*). Our data indicate that genetic deletion of NMDA receptor function in nNOS^+^ interneurons does not affect amplitude or kinetics of locomotion-alone–induced V1 astrocyte Ca^2+^ activation. This finding could be explained whether locomotion-induced noradrenergic terminal Ca^2+^ elevations did not rely on NO but were enhanced when more NO reaches the terminals during simultaneous visual input and locomotion. There is a 10-fold difference between the NO sensor soluble guanylate cyclase’s affinity for NO when it is measured with isolated protein compared to cellular preparations (*77*, *78*). Therefore, it is reasonable to assume that considerable differences in NO sensitivity among cell types exist. Alternatively, our finding could indicate that locomotion-induced nNOS^+^ interneuron Ca^2+^ elevations and subsequent nNOS activation are independent of NMDA receptor activation.

The noradrenergic terminal integration model, which we are proposing here, has several conceptual implications: (i) Despite the sparsity of nNOS^+^ interneuron somata, the axons form a dense network throughout the neocortex (*47*). Similarly, noradrenergic terminals are ubiquitous within the neocortex (*14*). Therefore, it is possible that the noradrenergic terminal integration model does not only apply to the visual cortex but may represent a general cortical circuit motif for the modality-selective integration of sensory input and vigilance state. (ii) It implies that astrocytes reliably track and amplify extracellular norepinephrine dynamics, which carry integrated information about sensory input and vigilance state, rather than integrating this information themselves. There is an independent finding in support of the idea that astrocytes reliably report norepinephrine dynamics. It was previously found that locomotion-induced Ca^2+^ elevations are faster and shorter in cerebellar Bergmann glia than in V1 astrocytes (*36*). Here, we revealed that the considerably faster locomotion-induced Ca^2+^ elevations in noradrenergic terminals in the cerebellum compared to those in V1 can provide an explanation for this difference in kinetics of astroglia Ca^2+^ dynamics. It is possible that the brain region–specific differences in vigilance-dependent noradrenergic terminal Ca^2+^ elevations are due to differences in LC neuron intrinsic excitability as has been found for LC neurons that target different forebrain areas (*17*). (iii) Last, the noradrenergic terminal integration model implies that integration of sensory information is encoded in local noradrenergic signaling through lower affinity α_1_- and β-adrenergic receptors on neurons, vasculature, and glia (*79*, *80*). Increases of noradrenergic tone in V1 can convert sensory input-dependent long-term depression of glutamatergic transmission into spike timing-dependent plasticity (*81*). Therefore, short-term potentiation of norepinephrine release could be part of a local feedback loop to further enhance signal to noise of sensory input, which is an important component of attentional deployment models (*3*). It has been found that exogenous, chemogenetic overactivation of striatal astrocyte Ca^2+^ signaling, which would override the reliable reporting of norepinephrine dynamics by astrocytes, led to hyperactivity and attention deficits in mice (*33*). Therefore, local integration of sensory information and vigilance state by noradrenergic terminals may represent a circuit component of a bottom-up mechanism for sensory modality-selective attention.

## MATERIALS AND METHODS

### Experimental design

All animal procedures were conducted in accordance with the guidelines and protocols of the University of Texas Health Science Center at San Antonio’s Institutional Animal Care and Use Committee. At the time of data collection, mice in most experiments were between 2 and 6 months old, except those for local pharmacology experiments (Figs. 4 and 5 and fig. S5) that were 2 to 3 months old to maximize the recovery from acute surgery. For all awake behavior experiments, we used a previously reported head-restrained mouse on a motorized treadmill paradigm (*36*). Locomotion protocols as described in the respective figures and subsequent data analysis routines were automated to minimize the risk of experimenter bias. On the basis of availability, male and female mice were assigned randomly to individual experiments, and all datasets contain data from mice of both sexes. Disaggregated data analysis did not reveal any trend that could be accounted for by a sex effect. For all experiments, the statistical analysis was based on the number of mice (at least six).

### Animals

Mice were kept in the Laboratory Animal Resources facility with ad libitum access to water and chow. Mice were maintained on a reverse 12-hour light/12-hour dark schedule (lights off at 9 a.m., lights on at 9 p.m.) and all experimental procedures were completed during the dark cycle and under dark conditions. For experiments in Figs. 1, 3, and 5 and figs. S2, S3, S4, and S5, GCaMP6f was expressed in astroglia using *Aldh1l1-CreER*^*T2* +/−^ (*42*);Ai95^+/−^ (*82*) mice (the Jackson Laboratory, stock nos. 031008 and 028865, respectively). For experiments in Figs. 2 and 4 and fig. S5, Lck-GCaMP6f was expressed in noradrenergic neurons using *Dbh-Cre*^+/−^ (*43*);*Lck-GCaMP6f* ^flox +/−^ (*42*) mice [Mutant Mouse Resource and Research Center (MMRRC), stock no. 036778-UCD; and the Jackson Laboratory, stock no. 029626, respectively]. For experiments in Fig. 2B, *Dbh-Cre*^+/−^;Ai14^+/−^ (*83*) mice were used (the Jackson Laboratory, stock no. 007914). For experiments in Fig. 6 and fig. S6, *nNOS-CreER*^*T2* +/−^ (*49*);c*GluN1*^flox/flox^ (*48*) or *nNOS-CreER*^*T2* +/−^;c*GluN1*^wt/wt^ littermates were used (the Jackson Laboratory, stock nos. 014541 and 005246, respectively). Experimenters were blinded regarding the allocation of floxed or wild-type mice to experimental groups.

### Tamoxifen administration

Tamoxifen (Sigma-Aldrich, no. T5648-5G) was freshly dissolved in sunflower seed oil (Sigma-Aldrich, no. 1642347-1G) at a concentration of 10 mg/ml by vortexing and sonication for approximately 15 min. For experiments using the *Aldh1l1-CreER^T2^* mouse line, tamoxifen was injected intraperitoneally (10 μl per gram of mouse body weight starting at age 3 to 4 weeks) at 100 mg/kg (fiber photometry experiments) or 75 mg/kg (two-photon microscopy experiments), using a 1-ml syringe and a 25-gauge 5/8-inch needle (BD PrecisionGlide Needle). Surgeries were performed 1 week after tamoxifen injection. To maximize Cre recombination in *nNOS-CreER*^*T2* +/−^;c*GluN1*^flox/flox^ mice, tamoxifen administration was extended for these mice and their wild-type littermates to five injections within 9 days.

### Animal surgery

Surgery procedures consisted of two steps. In the first-step surgery, under intraperitoneal anesthesia [ketamine (100 mg/kg) and xylazine (10 mg/kg) in 0.9% saline solution], the mouse was placed on a heating pad to keep body temperature at ~36°C. Following removal of the hair, the skin was disinfected using povidone-iodine. The skin and muscles were removed from the skull, and 3% hydrogen peroxide was applied to further aid in disinfection and to prevent bleeding. The periosteum was shaved off, and approximately 3 mm of muscle surrounding the exposed skull was covered with a thin layer of cyanoacrylate cement. For dual-site fiber photometry experiments, a custom-designed stainless steel head plate with a thin cross-bow shape was centered just anterior to the bregma suture (Figs. 1 and 3 and figs. S3 and S4); for two-photon imaging, a stainless steel head plate designed with a 4 mm–by–6 mm oval opening was centered on the skull above lobulus simplex/crus I of the cerebellar hemisphere (Fig. 2), or above V1 at lambda, 2.5 mm lateral from midline (Figs. 2 and 4 to 6 and figs. S5 and S6). The head plate was mounted on the skull using dental cement (C&B Metabond, Parkell Inc., Brentwood). While mice recovered from anesthesia, the wound edges were coated with Neosporin ointment, and the mouse was placed in a heated cage for recovery. In the second-step surgery, under isoflurane anaesthesia (1.5 to 2% v/v isoflurane in O_2_ with flow rate adjusted according to hindpaw pinch reflex) and on a heating pad, craniotomies were each performed on a skull area of 2.5 mm by 2.5 mm. The dura mater was removed and replaced with three fused layers of no.1 cover glass. Cerebellar cranial windows were implanted above lobulus simplex/crus I of the cerebellar hemisphere (Figs. 1 to 3 and figs. S3 and S4), while visual cortex windows were implanted above V1 at lambda, 2.5 mm lateral from the midline (Figs. 1 to 6 and figs. S3 and S6). For all cranial window placements into the skull, the edges of the cranial window glass were gently sealed by dental cement (Ortho-Jet Acrylic Resin, Lang). After 1 week of postsurgery recovery, mice were habituated to the linear treadmill and to the recording conditions for at least 20 min each during two separate sessions. All experiments except for local pharmacology experiments started at least 2 weeks following surgery. The local pharmacology experiments (Figs. 4 and 5 and fig. S5) were conducted at least 7 days after the first-step surgery and after completion of the two separate habituation sessions.

### Pharmacology

For local incubation with drugs through an acute cranial window, three times the typical concentration reported for slice preparation experiments was chosen to compensate for dilution through longer diffusion distances and continuing blood circulation. D-AP5 (catalog no. 0106, Tocris) or l-NMMA acetate (catalog no. 07716, Tocris) was dissolved in artificial cerebrospinal fluid (aCSF) at a final concentration of 1 mM and 600 μM, respectively. Oxygenated (95% O_2_, 5% CO_2_) aCSF was prepared with the following components (in millimolars, pH 7.4): 119 NaCl, 2.5 KCl, 1 NaH_2_PO_4_, 1.3 MgCl_2_, 2 CaCl_2_, 26.2 NaHCO_3_, and 11 C_6_H_12_O_6_ (dextrose). To optimize access of drugs to the brain while preserving integrity of the tissue following removal of the skull, we removed the dura. To minimize perfusion noise disturbance during individual imaging trials, we replaced the aCSF on the surface of V1 with fresh aCSF heated to 36° to 37°C every 10 min. To dampen tissue movement associated with the mouse walking on the treadmill, we cemented a 3 mm (rostrocaudal direction)–by–800 μm strip of no. 1 cover glass across the skull window. Two additional bits of no. 1 cover glass cut to 2 mm by 800 μm were attached to the center of the larger glass described above using ultraviolet (UV) curable optical adhesive. This procedure provided sufficient gentle pressure against the surface of the cerebellum to enable two-photon imaging during locomotion events while providing access for diffusion of drugs into the imaged tissue. The same field of view was imaged before and after bath application of drugs. Animals were head-fixed for no more than 45 min during a single imaging session.

### Immunohistochemistry

Under intraperitoneal anesthesia [ketamine (100 mg/kg) and xylazine (10 mg/kg) in 0.9% saline solution], mice were perfused by cardiac puncture with ice cold 4% paraformaldehyde (Polysciences Inc.) in 0.1 M phosphate-buffered saline (PBS). Brains were removed from the skull and immersed in the same fixative solution for 4 hours at 4°C. The brain tissue was kept in PBS containing 0.1% sodium azide at 4°C. Parasagittal whole-brain sections were prepared on a vibratome (VF-300-0Z, Precisionary Instruments). Whole-brain sections (thickness of 35 μm) were soaked in the 0.1 M PBS for 10 min and incubated for 3 hours in blocking solution with 0.1 M PBS, 5% normal goat serum (NGS) (Jackson ImmunoResearch Laboratories Inc.), and 1% Triton X-100 (Sigma-Aldrich). The next step included incubation with the primary antibodies diluted in the blocking solution containing 5% NGS in 0.1 M PBS with 0.5% Triton X-100 for 36 hours at 4°C. The primary antibodies were used at the following concentrations: chicken anti-enhanced GFP (1:1000; Thermo Fisher Scientific, no. A10262, polyclonal) and mouse anti-S100β [1:500; Thermo Fisher Scientific, no. MA1-25005, monoclonal (SH-B4)] (fig. S1) or rabbit anti-tyrosine hydroxylase (1:1000; Abcam, ab112, polyclonal) (Fig. 2B). Sections were washed four times with 0.1 M PBS containing 5% NGS for 10 min each and incubated for 4 hours at room temperature in 0.1 M PBS with 5% NGS with diluted appropriate fluorescence-conjugated secondary antibodies: Alexa Fluor 488–conjugated AffiniPure Goat Anti-Chicken immunoglobulin Y (IgY) (1:5000; Jackson ImmunoResearch, no. 103-545-155), Alexa Fluor 647–conjugated AffiniPure Goat Anti-Mouse IgG1 (1:5000; Jackson ImmunoResearch, no. 115-605-205) (fig. S1), or Alexa Fluor 647 AffiniPure Goat Anti-Rabbit IgG (H+L) (1:5000; Jackson ImmunoResearch, no. 111-605-144) (Fig. 2B). Before mounting on microscope glass slides, the sections were rinsed once with 0.1 M PBS containing 5% NGS and three times with 0.1 M PBS without NGS for 10 min each. The slices were mounted using aqua-poly/mount coverslipping medium (Polysciences Inc., no. 18606-20) and dried overnight. The next morning, they were sealed with two-part epoxy adhesive (Bob Smith Industries Inc., BSI-201).

### Confocal imaging

The confocal imaging data were obtained using a Zeiss LSM 710 confocal microscope. For whole-brain parasagittal sections, tiled scanning was used with a 10× objective [Plan-Apochromat, 0.45 numerical aperture (NA), Zeiss]; 5 *Z*-stack slices with 4-μm step size at <1 airy unit pinhole setting (fig. S1). For higher-magnification images, a 40× oil immersion objective (EC Plan-Neofluar, 1.3 NA, Zeiss) was used; 12 *Z*-stack slices with 0.45-μm step size at <1 airy unit pinhole setting (Fig. 2B and fig. S2). Images represent maximum intensity projections of image stacks. The 488-nm line of an argon laser was used to excite Alexa Fluor 488, while the 560- and 633-nm lines were used to excite TdTomato fluorescence and Alexa Fluor 647, respectively.

### Macroscopic photography

The photographs in fig. S1 were taken using an Olympus PEN-F digital camera mounted to a Leica S6D stereo microscope via a Leica 10445929 0.5× adapter.

### Dual-site Fiber photometry

To achieve simultaneous fluorescence recordings from both the cerebellum and V1, two individual multimode fibers (MMF, 400-μm core diameter, 0.5 NA, FP400URT, Thorlabs) were positioned at the surface of the cranial windows above cerebellum and V1, respectively (Fig. 1A). Fiber optics were inserted into a titanium tube (New England Small Tube Corporation, no. PB09125) held at the cranial window glass surface by a translation stage and clamp apparatus (Thorlabs Inc., no. MT1/M) to ensure stability during in vivo experimentation. To maintain a good optical coupling for excitation and emission transmission through the cranial window, a drop of immersion oil (Zeiss, no. 4449600000000) was injected both at the base of the titanium tube and into the titanium tube’s top opening before fiber insertion. The fibers were then each inserted into the titanium tubes held above respective cranial windows of cerebellum and V1. A ring of moldable clay (Michael’s Crafts, no. 10062488) encircled both cranial windows and layered pieces of black felt (Michael’s Crafts, no. D006440S) were stacked above the cranial windows both to prohibit optical cross-talk between the neighboring fiber-optic terminals and to avoid stray laser light scattering from each glass cranial window. Excitation light from a blue diode laser (460 nm, MDL-III-460, OptoEngine LLC) was reflected off a dichroic mirror (FF495-Di03-25x36, Semrock) and coupled into the anterior ends of the two fibers. Anterior ends of the fiber optics were held in place by a physical contact fiber-optic connector (FC/PC Multimode Connector, 850-μm bore, Thorlabs) that was connected to a cage plate with a fiber adaptor (Fiber Adapter Plate with External SM1 Threads, SM1FC, Thorlabs Inc.) and cage cube (30-mm cage cube, C6, Thorlabs Inc.) setup that housed the dichroic mirror. Excitation power output from fiber distal ends was continuous and adjusted to be approximately 200-μW output power from each fiber. Emission signal from GCaMP6f was collected through each fiber-optic and delivered through the dichroic mirror to the detection system which included a 10× infinity-corrected objective lens (×10 magnification, 0.28 NA, 34.0-mm working distance, Mitutoyo), an emission filter (BLP01-473R-25, Semrock), and a machine vision lens (MVL50M23, Thorlabs) mounted with a 10-mm extension tube to shorten the minimal focal distance (macroeffect) to a USB-CMOS camera (BFLY-PGE-31S4M-C, PointGrey). Camera settings were controlled in NI-MAX (National Instruments Measurement and Automation Explorer, Version 17.0, National Instruments) on an Xi Computer MTower PCIe workstation (Intel(R) Core i7-4930K CPU @ 3.40 GHz, 8 GB of RAM). The camera acquired images through external triggering via a custom-written script in LabVIEW (LabVIEW 2013, National Instruments). Images (16 bit, 200 × 290 pixels) of the fiber-optic ends were acquired at a frame rate of 20.5 frames/s with an acquisition gain of 20 dB and exposure time of 10 ms. A 2 × 2 binning was used with pixel averaging. The entire experimental setup was enclosed in a blackout box. For the cells responsible for the cerebellar signal, we use the term “cerebellar astroglia” because fiber photometry excites fluorophores and collects fluorescence from a volume rather than a precise optical plane and may contain fluorescence signals from molecular layer Bergmann glia processes and granular layer velate astrocytes. However, photometry signals drop steeply with axial distance from the fiber facet (*84*, *85*). Therefore, with the molecular layer closest to the fiber facet and velate astrocytes at a lower density among the densely packed granule cells, we can assume that Bergmann glia contributed the vast majority of the cerebellar fluorescence signal.

### In vivo two-photon imaging

For all two-photon experiments (Figs. 2 and 4 to 6 and figs. S5 and S6), we used a resonant scanning version of the Movable Objective Microscope (Sutter Instruments) with a 16×, 0.80 NA water immersion objective (Nikon). A pulsed Ti:Sapphire laser beam at 920 nm, 80-MHz repetition rate, and <120-fs pulse width (Insight DS+, Spectra-Physics MKS Instruments Light & Motion) was used for two-photon excitation. The image acquisition rate was 30 frames/s with the laser power adjusted to 25 to 32 mW at the front aperture of the objective. The microscope was controlled by an Xi Computer MTower PCIe workstation [Intel(R) Core i7-5930K CPU @ 3.50 GHz, 16 GB of RAM] running ScanImage (v5.5; Vidrio Technologies, LLC) software within MATLAB R2019a (MathWorks). We focused at approximately 60 μm below the pial surface reaching half-way through the cerebellar molecular layer or deep into layer 1 of V1. Emitted light was detected using a GaAsP photomultiplier tube (Hamamatsu Photonics, H10770PA-40). All data were acquired using volumetric scanning (Physik Instrumente, PD72Z4CAA) with a step size of 10 μm, covering 40 μm in the *z* plane, at a frequency of 5 volumes/s. Acquired frames were 400 μm by 400 μm at a resolution of 512 pixels per line and 512 lines per frame. The entire experimental setup was enclosed in a blackout box.

### Locomotion behavioral paradigm

Awake mice were placed on a custom-made linear treadmill (*36*). The speed of the treadmill belt was monitored with an optical encoder (Honeywell, no. 600-128-CBL). The belt was freely movable so that the mouse could walk voluntarily; however, at predefined episodes a servo motor was engaged to enforce locomotion at 80 to 110 mm/s. National Instruments boards controlled by custom written scripts in LabVIEW 2013 (version 13.0.1f2, National Instruments) were used to trigger image acquisition and for simultaneous recording at 20-kHz sampling rate of locomotion speed data and Y-mirror position (two-photon imaging) or CMOS camera frame timestamp (fiber photometry) data. Nonimaging data were post hoc downsampled to the image acquisition frame rate and the Y-mirror or frame timestamp data were used to assign appropriate data bins to individual image frames.

### Visual stimulation

Visual stimulation during in vivo experiments consisted of a natural scene image of a close-up plot of grass that was selected from the van Hateren’s Natural Image Dataset (image 0600; http://bethgelab.org/datasets/vanhateren/) and presented to mice during experiments. The natural scene visual stimulus represented a 5-s-long AVI movie (100 identical images at 20 frames/s) and was displayed on a monitor [7-inch liquid crystal display (LCD), 864 × 480 pixels, KUMAN] connected to a Raspberry Pi (Model 3B+, Raspberry Pi Foundation). The monitor was centered and positioned 10 cm away from the mouse to achieve uniform exposure to the visual stimulus. For costimulation trials, the movie was played during enforced locomotion. To avoid light interference from visual stimulation presentation with the two-photon detection system during two-photon microscopy experiments, a KODAK Wratten 2 no. 47 (deep blue) filter (Edmund Optics, no. 53-700; fig. S4A) was attached to the display monitor screen. Dual-site fiber photometry experiments did not require covering the monitor screen with a filter unless performed for evaluation of this approach (fig. S4). Pulse-width modulation of the display monitor was used to control the timing of the visual stimulus presentation and to limit the display’s luminance to 0.3 cd/m^2^ during low-intensity stimulation and 2.2 cd/m^2^ during high-intensity stimulation in dual fiber experiments, while the blue filter reduced the low-intensity stimulation to 0.1 cd/m^2^. Illuminance was measured via an illuminance meter (Mavolux 5032C/B USB, Gossen). Power output was measured with a power meter (FIELDMAXII-TOP, Coherent) using a high-sensitivity optical sensor (OP-2 VIS, active area diameter of 7.9 mm, Coherent). During natural scene display without blue filter the power output was 0.92 to 1.01 nW per mouse eye (pupil diameter of 4 mm in darkness) during low-intensity stimulation, 7.23 to 7.59 nW per mouse eye during high-intensity stimulation, and 0.19 to 0.35 nW per mouse eye during low-intensity stimulation with blue filter. Spectrum recordings shown in fig. S4 were acquired with a USB series UV-VIS spectrometer (USB 2000+ UV-VIS, Ocean Optics).

### AAV preparation

To specifically express GCaMP3 in astroglia of *nNOS-CreER*^*T2* +/−^;c*GluN1*^flox/flox^ or *nNOS-CreER*^*T2* +/−^;c*GluN1*^wt/wt^ mice (Fig. 6), we replaced mKate2.5f within the AAV-GFAP-mKate2.5f plasmid (a gift from V. Gradinaru, California Institute of Technology, Pasadena, CA; Addgene, 99129) with GCaMP3. GCaMP3 was polymerase chain reaction–amplified out of the Zac2.1 gfaABC1D-Cyto-GCaMP3 plasmid (a gift from B. Khakh; Addgene, 44331) by adding a 5′-AflII and a 3′-BamHI restriction digestion site. AAV-gfaABC1D-GCaMP3 was then packaged into the AAV-PHP.B capsid (a gift from V. Gradinaru; Addgene, 103002) by the University of Pennsylvania Vector core.

### AAV delivery

To facilitate blood-brain-barrier passage (*86*), we freshly prepared d-mannitol (VWR, 0122-500G) with 0.9% saline for a final concentration of 25% d-mannitol solution (heating the solution at ~70°C for 5 min helped d-mannitol to dissolve). Twenty minutes before the viral vector delivery, d-mannitol solution was applied to the animals by intraperitoneal injection (30 μl/g mouse body weight). AAV-PHP.B-gfaABC1D-GCaMP3 was diluted in Dulbecco’s PBS (Gibco, 14190-144). The viral vector (50 μl; 1.4 × 10^11^ genome copies) was then delivered via an insulin syringe (EasyTouch U-100, 830365; 0.3 ml, 30 gauge × 5/16 inches) through free-hand retro-orbital intravenous sinus injection under isoflurane anesthesia. As our imaging experiments were performed on the left hemisphere of the brain, viral vectors were injected through the left retro-orbital sinus of the animals to avoid damaging the respective visual input. Animals were returned to the animal holding room after they recovered from anesthesia.

### Data analysis

Data from dual-site fiber photometry recordings were averaged (every four consecutive image frames), to result in a final effective frame rate of 5.0 Hz. ROIs representing imaged endings of fibers that were placed above Cb or V1 were defined in averaged time series images (Fig. 1A). We determined the median fluorescence value during baseline (from start of a trial until the frame before onset of locomotion) and then calculated the Δ*F*/*F* fluorescence values for each image frame by (*F* − *F*_median_)/*F*_median_, with *F* being the absolute mean fluorescence value within a given ROI in a given image frame. For all dual-site fiber photometry data, the term “mean Ca^2+^ change” represents mean Δ*F*/*F* from onset of locomotion to the peak of the transient response.

Imaging data collected from two-photon microscopy experiments were saved in ScanImage as tiff files, imported to MATLAB R2016a and stored as .mat files. Analysis was conducted using custom-written scripts using a combination of built-in, open-source, and custom-written functions. Any computer code will be shared upon request. A maximum intensity projection was applied to each scanned volume to obtain a more complete representation of individual ROI Ca^2+^ dynamics. Images were first passed through a Gaussian filter (1.52 SD per pixel distance) to attenuate random noise of the detector, and individual frames within the entire time series (including all imaging trials, one trial representing baseline, 5-s locomotion episode followed by imaging episode until the next imaging pause) were registered to maximize correlation. For noradrenergic terminal imaging, where individual processes expressing membrane-anchored GCaMP6f could not be reliably discerned, ROIs were spatially defined by an 8 by 8 checkerboard pattern, which resulted in ROIs of approximately 50 μm by 50 μm (Figs. 2 and 4 and fig. S5). For individual V1 astrocytes (Figs. 5 and 6 and figs. S5 and S6), ROIs were drawn over individual astrocyte somata and surrounding processes. For each ROI, the photomultiplier tube offset that was determined with the laser turned off was subtracted from the fluorescence time series, which was determined as mean fluorescence signal within the ROI within each consecutive maximum intensity projection. Fluorescence dynamics within individual ROIs were then expressed as Δ*F*/*F*, calculated as (each maximum intensity projection fluorescence value − median fluorescence value during baseline before the locomotion)/median fluorescence value during baseline before the locomotion. For all two-photon microscopy data, the term mean Ca^2+^ change represents mean Δ*F*/*F* within a 10-s period beginning with the onset of locomotion.

We previously found that voluntary locomotion-induced astroglial Ca^2+^ elevations during the baseline episodes can attenuate enforced locomotion-induced responses (*36*). Therefore, in both two-photon Ca^2+^ imaging and dual-site fiber photometry, we determined the average SD of baseline Δ*F*/*F* values of all trials of a dataset. We then used the criterion if two consecutive Δ*F*/*F* values within a baseline exceeded 3× SD, this trial was considered “contaminated” by voluntary locomotion, and it was excluded from quantification.

It should be noted that any change in baseline fluorescence will influence the calculated Δ*F*/*F* fluorescence values. Table S1 summarizes the average median fluorescence values during baseline of locomotion-alone trials versus respective costimulation trials of all datasets. Changes in median fluorescence values during baseline were not significantly different and could not account for the effect of visual input studied here.

### Statistical analysis

Statistical analyses were performed using MATLAB R2016a (MathWorks). For each group of a dataset, the Lilliefors test was applied to test for Gaussian distribution. If all groups followed a Gaussian distribution, then we applied repeated-measures analysis of variance (ANOVA), followed by Tukey-Kramer correction for multiple comparisons on one group (Figs. 1 and 3 and fig. S4), on two groups (Fig. 6 and fig. S3), or on three groups (Figs. 4 and 5); unpaired, two-tailed Student’s *t* test was applied in figs. S5 and S6, followed by Bonferroni correction for multiple comparisons in fig. S5. If any group did not follow a Gaussian distribution, then we applied Wilcoxon signed-rank test, followed by Bonferroni correction for multiple comparisons (Figs. 2 and 4). All analyzed comparisons are labeled with the respective *P* value with a significance level of 0.05. The basis of the sample number for individual tests and the respective test type applied are mentioned in the figure legends.

## Supplementary Materials

### This PDF file includes:

Figs. S1 to S6 Table S1 Legends for Data files S1 to S10 Legend for Software code for data analysis and display

### Other Supplementary Material for this manuscript includes the following:

Data files S1 to S10

Software for data analysis and display

## Notes

View/request a protocol for this paper from *Bio-protocol*.
