# Supplementary material for: Noradrenergic terminal short-term potentiation enables modality-selective integration of sensory input and vigilance state
Source: Sci Adv. 2021 Dec 17;7(51):eabk1378. doi: 10.1126/sciadv.abk1378 (PMC8682997; doi:10.1126/sciadv.abk1378)
Supplement: Supplementary file 1 — Figs. S1 to S6 Table S1 Legends for Data files S1 to S10 Legend for Software code for data analysis and display [file sciadv.abk1378_sm.pdf]

Science Advances
AAAS

Supplementary Materials for

Noradrenergic terminal short-term potentiation enables modality-selective integration of sensory input and vigilance state

Shawn R. Gray, Liang Ye, Jing Yong Ye, Martin Paukert*

*Corresponding author. Email: paukertm@uthscsa.edu

Published 17 December 2021, *Sci. Adv.* **7**, eabk1378 (2021)
DOI: 10.1126/sciadv.abk1378

The PDF file includes:

Figs. S1 to S6
Table S1
Legends for Data files S1 to S10
Legend for Software code for data analysis and display

Other Supplementary Material for this manuscript includes the following:

Data files S1 to S10
Software for data analysis and display

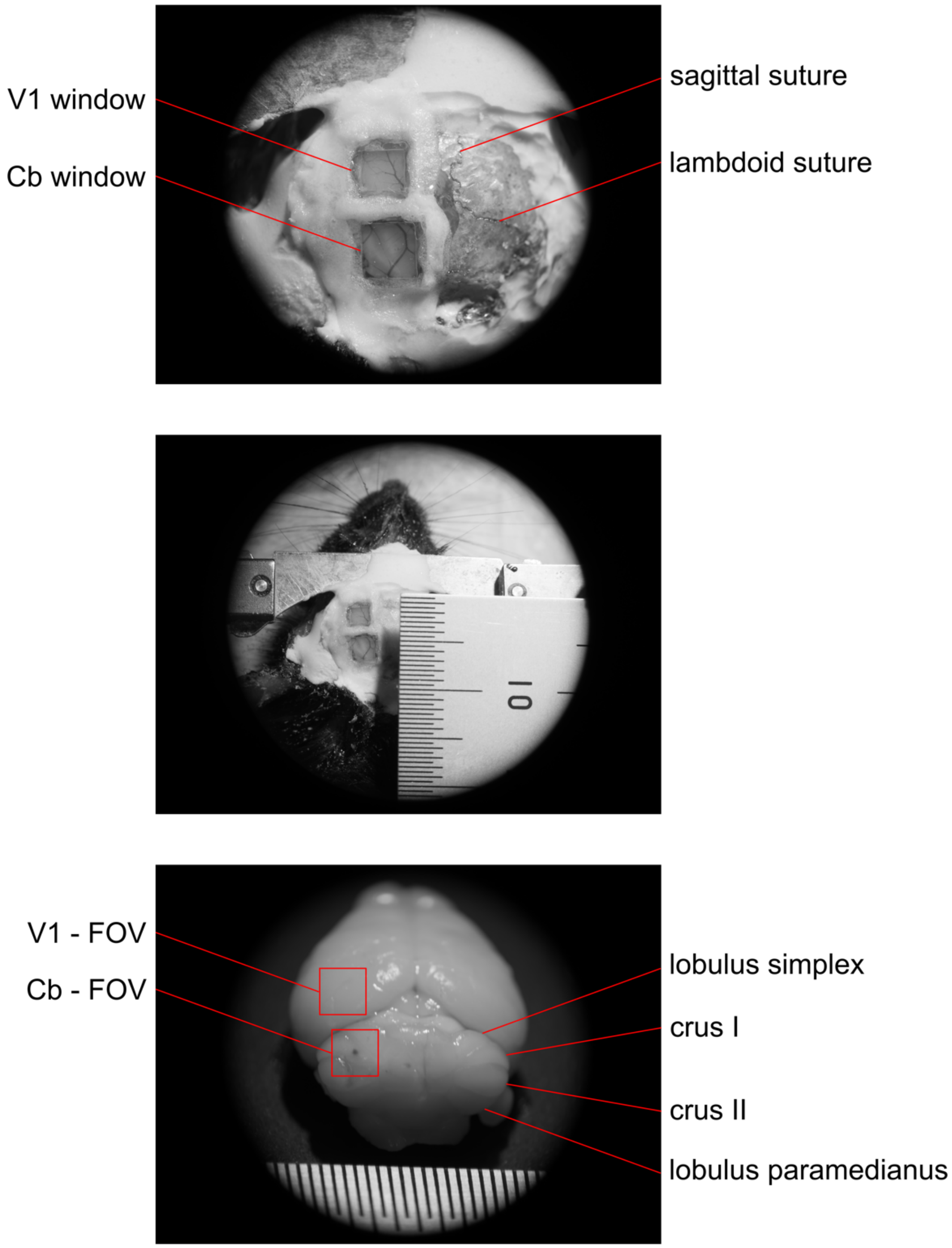

Fig. S1. Placement of chronic cranial windows for dual fiber photometry *(related to Fig. 1)*. **Upper**, chronic cranial windows above left hemisphere's primary visual cortex (V1) and cerebellar crus I and lobulus simplex (Cb) of mouse at 10 weeks of age. **Middle**, same view at lower magnification with scale bar (smallest divisions represent 500 µm). **Lower**, forebrain and cerebellum extracted from same mouse following paraformaldehyde intracardial perfusion fixation 3 days after chronic cranial window installation. Areas under respective windows and cerebellar anatomical landmarks are highlighted. Photo Credit: Martin Paukert, UTHSCSA

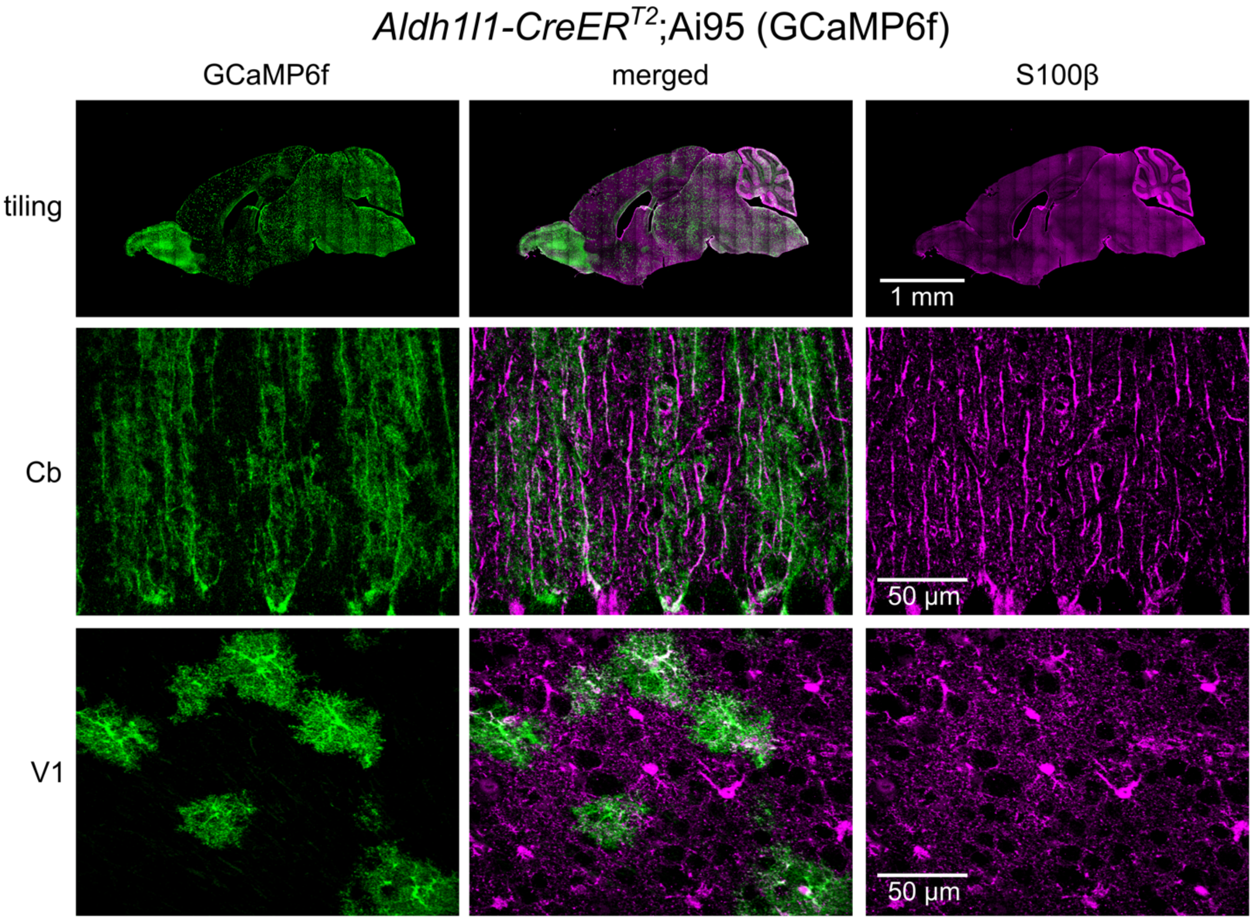

Fig. S2. Homogeneous, astroglia-specific GCaMP6f expression in *Aldh1l1-CreER^{T2}*;Ai95 mouse *(related to Fig. 1)*. **Upper**, whole-brain parasagittal section immunostained for eGFP (GCaMP6f; green) and S100β (magenta) from 12 week-old *Aldh1l1-CreER^{T2}*;Ai95 mouse, treated with 100 mg/kg i.p. injection of tamoxifen at the age of 3 weeks. Note, the olfactory bulb is the only brain region where GCaMP6f expression is visibly not restricted to astroglia. **Middle**, high magnification section in cerebellum (Cb); **lower**, high magnification section in primary visual cortex (V1).

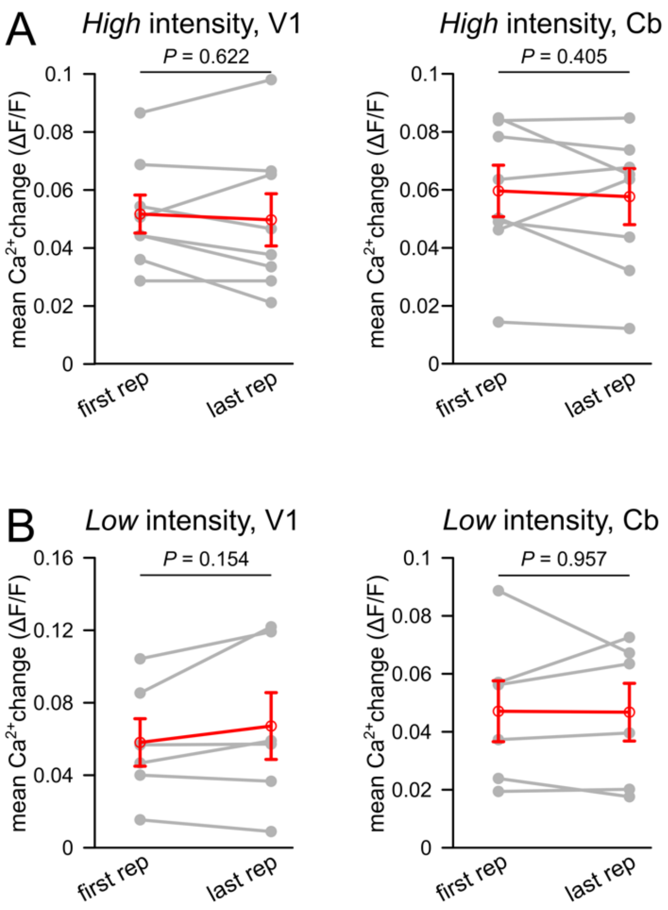

Fig. S3. Locomotion-induced astoglia Ca^{2+} activation is stable during repeated events *(related to Fig. 1)*. **(A)** For experiments in Fig. 1B where the effect of high intensity visual stimulation was tested, if more than one locomotion alone trial was "uncontaminated" by voluntary locomotion, the mean Ca^{2+} change in V1 (left) or Cb (right) of the first "uncontaminated" locomotion alone trial was compared to the last "uncontaminated" locomotion alone trial ($n = 8$). Gray lines connect values from the same mouse. Red symbols indicate mean ± SEM. Statistical analysis used repeated measures ANOVA for two groups (V1 and Cb), followed by Tukey-Kramer correction. **(B)** Same analysis as in (A), but for data of Fig. 1D where the effect of low intensity visual stimulation was tested ($n = 6$). Gray lines connect values from the same mouse. Red symbols indicate mean ± SEM. Statistical analysis used repeated measures ANOVA for two groups (V1 and Cb), followed by Tukey-Kramer correction. Source data are provided as a source data file.

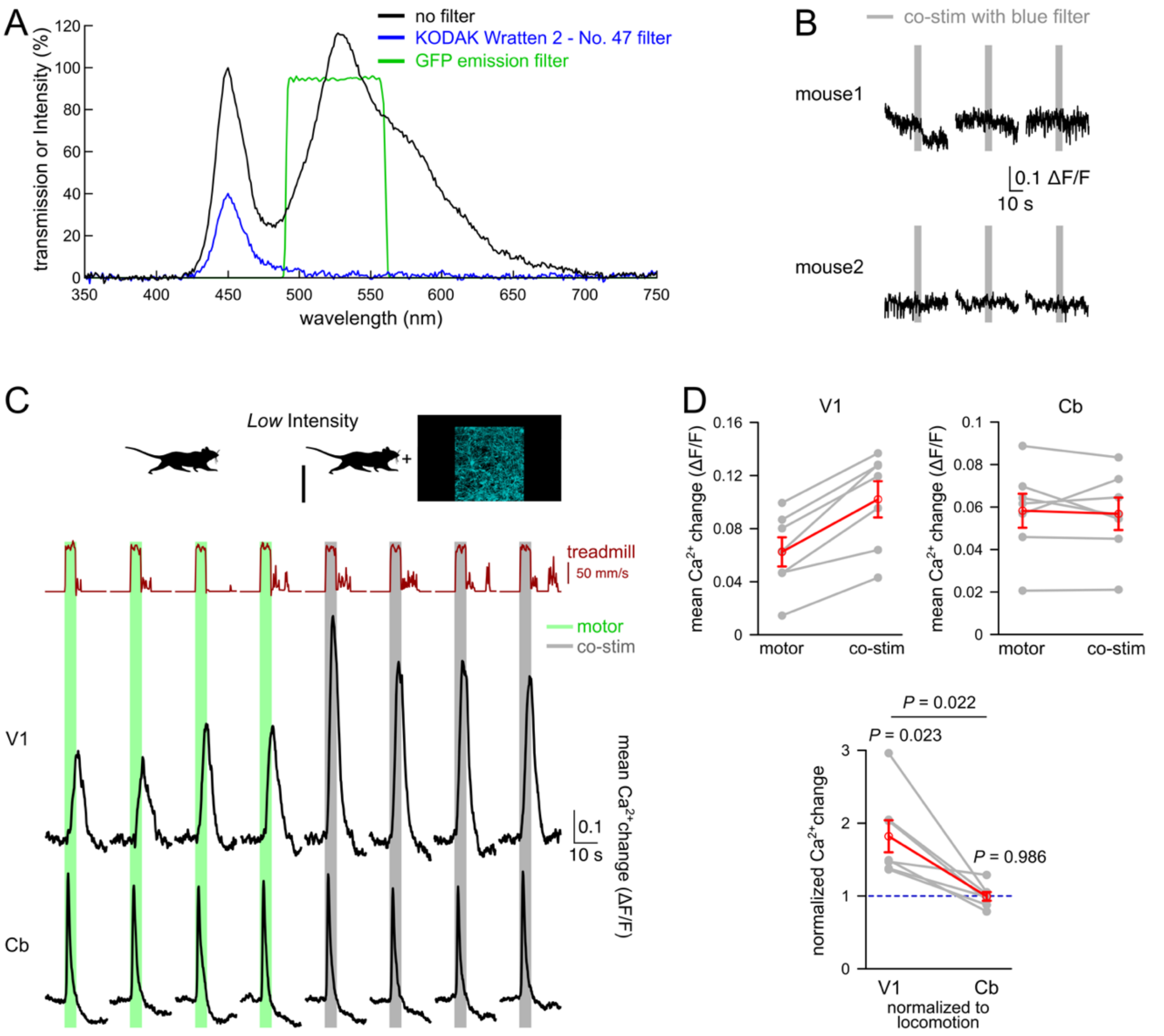

Fig. S4. Blue filtering of LCD monitor emission prevents leakage into two-photon GCaMP6f signal detection and reliably induces region-specific potentiation of vigilance-dependent V1 astrocyte Ca^{2+} activation, as evaluated with dual-fiber photometry *(related to Fig. 2)*. **(A)** Transmission/emission spectra: green trace, GFP emission filter (ET525/70m-2p, Chroma Technology, Corp.), used for two-photon detection of GCaMP6f signals; black trace, intensity of the LCD monitor emission without filter normalized to its peak near 450 nm; blue trace, intensity of the LCD monitor emission with KODAK Wratten 2, No. 47 (deep blue) filter normalized to the peak near 450 nm without filter. **(B)** Examples of two-photon fluorescence signals in cerebellum molecular layer of *GCaMP6f*^{negative}*;Aldh1l1-CreER*$^{T2\ +/-}$ mice, using the GFP emission filter from (A). The same GaAsP detector gain was used as in all two-photon microscopy experiments in this study. Gray bars, co-stimulation of locomotion and low-intensity blue visual stimulation (0.1 cd/m^2). **(C)** Fiber photometry Ca^{2+} dynamics in V1 astrocytes (V1) and cerebellar astroglia (Cb) in *Aldh1l1-CreER*^{T2};Ai95 mice during locomotion (green bars) or co-stimulation (gray bars). **(D) Upper**, Population data of mean Ca^{2+} elevations during locomotion or co-stimulation in V1 or Cb. **Lower**, Ca^{2+} elevations during co-stimulation normalized to respective locomotion response in both regions ($n = 7$ mice). Gray lines, same mouse. Red symbols, mean ± SEM. Repeated measures ANOVA followed by Tukey-Kramer correction. Individual *P*-values represent comparisons to 1 (blue dashed line), respectively. Source data are provided as a source data file.

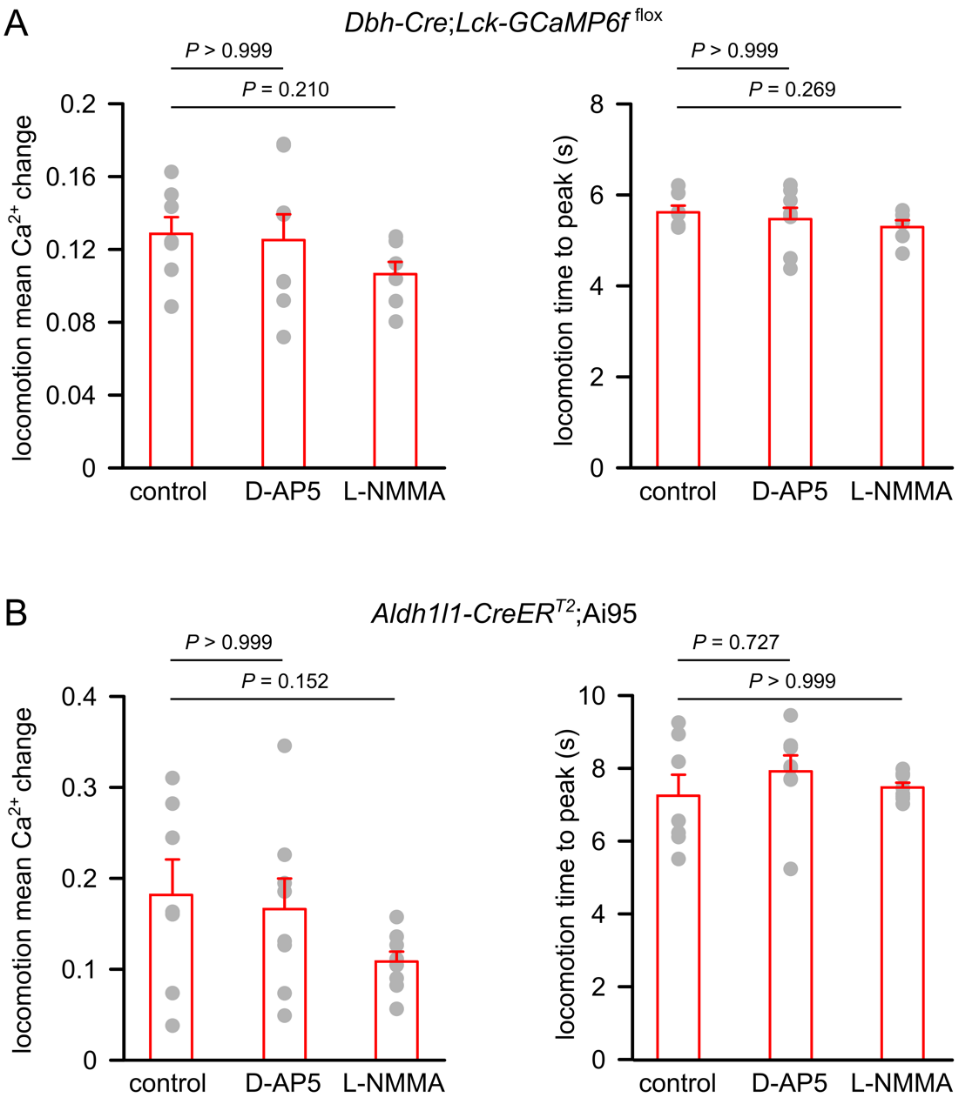

Fig. S5. Neither amplitude nor kinetics of locomotion alone-induced Ca^{2+} responses in V1 noradrenergic terminals or astrocytes were affected by D-AP5 or L-NMMA acetate *(related to Figs. 4 and 5)*. **(A)** Scattered dot plots represent mean Ca^{2+} change (left) and mean time to peak (right) in V1 noradrenergic terminals from Fig. 4 in response to locomotion alone during incubation with aCSF (n = 7 mice), D-AP5 (n = 8 mice) or L-NMMA acetate (n = 6 mice). Red bars and whiskers represent mean ± SEM. Statistical analysis used unpaired, two-tailed Student's t-tests for indicated comparisons followed by Bonferroni correction. **(B)** Same analysis but for V1 astrocytes from Fig. 5 in response to locomotion alone during incubation with aCSF (n = 7 mice), D-AP5 (n = 8 mice) or L-NMMA acetate (n = 8 mice). Red bars and whiskers represent mean ± SEM. Statistical analysis used unpaired, two-tailed Student's t-tests for indicated comparisons followed by Bonferroni correction. Source data are provided as a source data file.

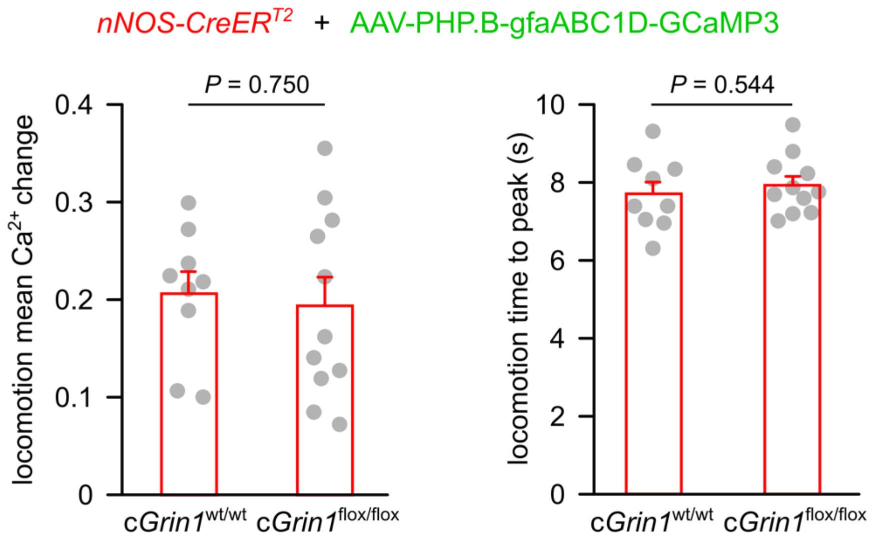

Fig. S6. Amplitude and kinetics of Ca^{2+} responses to locomotion alone were not affected by conditional gene deletion of GluN1 in nNOS^{+} interneurons *(related to Fig. 6)*. Bar graphs compare locomotion-induced mean Ca^{2+} change (left) and mean time to peak (right) in V1 astrocytes between *nNOS-CreER^{T2}*;c*Grin1*$^{wt/wt}$ (*n* = 9 fields of view, 3 mice) and *nNOS-CreER^{T2}*;c*Grin1*$^{flox/flox}$ (*n* = 11 fields of view, 4 mice) with mice from Fig. 6. Red bars and whiskers represent mean ± SEM. Statistics were conducted by unpaired, two-tailed Student's *t*-test. Source data are provided as a source data file.

median baseline fluorescence values

data	mean ± SEM		counts (*n*)	*P*-values
	motor (a.u.)	co-stim (a.u.)		
Fig. 1 (V1)	337.860 ± 2.654	329.406 ± 2.215	15	0.512
Fig. 1 (Cb)	640.899 ± 2.776	632.494 ± 1.866	15	0.514
Fig. 2 (V1)	196.629 ± 0.651	198.825 ± 0.712	15	0.337
Fig. 2 (Cb)	182.012 ± 0.764	182.157 ± 0.819	15	0.949
Fig. 3 (V1)	357.468 ± 2.776	361.349 ± 1.983	7	0.378
Fig. 3 (Cb)	596.899 ± 3.327	609.479 ± 3.669	7	0.059
Fig. 4	208.959 ± 0.437	209.641 ± 0.403	21	0.594
Fig. 5	84.430 ± 0.255	85.470 ± 0.202	26	0.403
Fig. 6	89.664 ± 0.431	89.912 ± 0.355	20	0.155
Fig. S2 (V1)	320.557 ± 1.567	314.145 ± 2.746	7	0.255
Fig. S2 (Cb)	670.211 ± 4.657	661.556 ± 4.256	7	0.112

Table S1. Median baseline fluorescence values *(related to indicated figures)*. These values were used to calculate ΔF/F ((fluorescence - median baseline fluorescence) / median baseline fluorescence) values. Statistical analysis used paired, two-tailed Student's *t*-test. Since none of the comparisons reached the significance level of $P < 0.05$, we abstained from corrections for multiple comparisons. a. u., arbitrary unit (absolute detector voltage signal [mV] following amplification, filtering and digitization).

Data S1. Source data for Fig. 1.

Data S2. Source data for Fig. 2.

Data S3. Source data for Fig. 3.

Data S4. Source data for Fig. 4.

Data S5. Source data for Fig. 5.

Data S6. Source data for Fig. 6.

Data S7. Source data for Fig. S3.

Data S8. Source data for Fig. S4.

Data S9. Source data for Fig. S5.

Data S10. Source data for Fig. S6.

abk1378_software.zip. Software code for data analysis and display.
